# Immunogenicity and Protective Potential of Mucosal Vaccine Formulations Based on Conserved Epitopes of Influenza A Viruses Fused to an Innovative Ring Nanoplatform in Mice and Chickens

**DOI:** 10.3389/fimmu.2021.772550

**Published:** 2021-11-11

**Authors:** Cynthia Calzas, Molida Mao, Mathilde Turpaud, Quentin Viboud, Joelle Mettier, Thomas Figueroa, Pierre Bessière, Antoine Mangin, Laura Sedano, Pierre-Louis Hervé, Romain Volmer, Mariette F. Ducatez, Steve Bourgault, Denis Archambault, Ronan Le Goffic, Christophe Chevalier

**Affiliations:** ^1^ Institut National de Recherche pour l’Agriculture, l’Alimentation et l’Environnement (INRAE) Molecular and Virology Unit VIM-Unité Mixte de Recherche (UMR) 892, University Paris-Saclay, Jouy-en-Josas, France; ^2^ Institut National de Recherche pour l’Agriculture, l’Alimentation et l’Environnement (INRAE) Unité Mixte de Recherche (UMR1225), Interactions Hótes-Agents Pathogénes-Ecole Nationale Vétérinaire de Toulouse (IHAP-ENVT)-University of Toulouse, Toulouse, France; ^3^ Dementia Research Institute, Cardiff University, Cardiff, United Kingdom; ^4^ Chemistry Department, Université du Québec à Montréal, Montreal, QC, Canada; ^5^ Department of Biological Sciences, Université du Québec à Montréal, Montreal, QC, Canada

**Keywords:** influenza A viruses, highly pathogenic avian influenza virus, mucosal vaccines, adjuvants, nanoparticles, M2e/HA2 subunit vaccines

## Abstract

Current inactivated vaccines against influenza A viruses (IAV) mainly induce immune responses against highly variable epitopes across strains and are mostly delivered parenterally, limiting the development of an effective mucosal immunity. In this study, we evaluated the potential of intranasal formulations incorporating conserved IAV epitopes, namely the long alpha helix (LAH) of the stalk domain of hemagglutinin and three tandem repeats of the ectodomain of the matrix protein 2 (3M2e), as universal mucosal anti-IAV vaccines in mice and chickens. The IAV epitopes were grafted to nanorings, a novel platform technology for mucosal vaccination formed by the nucleoprotein (N) of the respiratory syncytial virus, in fusion or not with the C-terminal end of the P97 protein (P97c), a recently identified Toll-like receptor 5 agonist. Fusion of LAH to nanorings boosted the generation of LAH-specific systemic and local antibody responses as well as cellular immunity in mice, whereas the carrier effect of nanorings was less pronounced towards 3M2e. Mice vaccinated with chimeric nanorings bearing IAV epitopes in fusion with P97c presented modest LAH- or M2e-specific IgG titers in serum and were unable to generate a mucosal humoral response. In contrast, N-3M2e or N-LAH nanorings admixed with Montanide™ gel (MG) triggered strong specific humoral responses, composed of serum type 1/type 2 IgG and mucosal IgG and IgA, as well as cellular responses dominated by type 1/type 17 cytokine profiles. All mice vaccinated with the [N-3M2e + N-LAH + MG] formulation survived an H1N1 challenge and the combination of both N-3M2e and N-LAH nanorings with MG enhanced the clinical and/or virological protective potential of the preparation in comparison to individual nanorings. Chickens vaccinated parenterally or mucosally with N-LAH and N-3M2e nanorings admixed with Montanide™ adjuvants developed a specific systemic humoral response, which nonetheless failed to confer protection against heterosubtypic challenge with a highly pathogenic H5N8 strain. Thus, while the combination of N-LAH and N-3M2e nanorings with Montanide™ adjuvants shows promise as a universal mucosal anti-IAV vaccine in the mouse model, further experiments have to be conducted to extend its efficacy to poultry.

## Introduction

Avian influenza A viruses (AIV) remain one of the most important respiratory pathogens in humans and various animal species, including poultry. According to the disease severity in poultry, AIV can broadly be categorized as either highly pathogenic (HPAIV) or low pathogenic (LPAIV) AIV. In poultry, infection with LPAIV is asymptomatic or causes low to mild pathophysiological damages to the respiratory, digestive, and reproductive systems (resulting in a drop in egg production), while infection with HPAIV is characterized by high morbidity and mortality rates. AIV can not only dramatically impact the poultry industry by causing severe economic losses, but can also pose a serious threat to public health worldwide through their high rates of zoonotic infection and pandemic potential (1, 2).

Vaccination is the most efficient and cost-effective approach to protect human and animal populations against IAV. Current vaccines are primarily designed to generate an immune response directed towards surface hemagglutinin (HA) (especially the immunodominant HA head domain HA1) or neuraminidase (NA) glycoproteins. However, circulating IAV are continuously evolving, leading to the emergence of strains with new antigenic properties. Point mutations occurring in the HA protein caused by the error-prone viral RNA-dependent RNA polymerase (“antigenic drift”) lead to strains which can escape pre-existing host immune responses. In addition, the segmented nature of the viral genome allows for exchanges of viral segments in host cells co-infected with distinct IAV strains, resulting in reassortant viruses with new combinations of HA or NA surface glycoproteins (“antigenic shift”). These genetic reassortments could contribute to the emergence of novel IAV subtypes with pandemic potential (3). These antigenic drift and shift events occurring in circulating IAV may strongly reduce the efficiency of influenza vaccines (4, 5).

Owing to the high variability of IAV, an active area of research focuses on developing subunit vaccines containing conserved “universal” viral antigens, including epitopes located in the membrane proximal stalk domain of HA (HA2) and the ectodomain of the matrix protein 2 (M2e) (4, 6–8). Multiple vaccine candidates and platforms incorporating HA2 and/or M2e epitopes are under development, including fusion proteins with bacterial enterotoxins, flagellin, *Neisseria meningititis* outer membrane protein complex or *Mycobacterium tuberculosis* heat-shock protein 70, virus-like particles, bacterial outer membrane vesicles, bacteriophages, liposomes, immune stimulating complexes (ISCOM), bacterial or viral vectored vaccines, DNA or RNA vaccines, organic polymers such as chitosan, poly(lactic-*co*-glycolic acid) or poly-γ-glutamic acid particles and inorganic nanoparticles such as gold nanoparticles (5, 7–15). These vaccine candidates were shown to provide protection against homologous and/or heterologous IAV challenges in pre-clinical trials. Whereas anti-HA1 antibodies block viral entry into host cells by inhibiting the interaction between HA1 and sialic acid residues on cellular receptors, anti-HA2 antibodies neutralize infection at other stages of the virus life cycle, including the fusion step between viral and endosomal membranes. The HA2-specific antibodies also operate by engaging Fc-mediated effector functions including antibody-dependent cellular phagocytosis (ADCP) or antibody-dependent cell-mediated cytotoxicity (ADCC) (4, 6, 8). Vaccines incorporating a linear contiguous fragment of HA2, the long alpha helix (LAH), elicited protective LAH-specific humoral responses in mouse studies (16–18). M2e is sparsely expressed at the surface of the virus but is abundant at the surface of infected cells. M2e-specific antibodies are non-neutralizing but exhibit Fc-mediated protective effector functions (4, 6–8). The protective potential of HA2- and M2e-based vaccines also correlates with the generation of specific cell-mediated immune responses (7, 8). Although vaccine trials with HA2- or M2e-based formulations gave encouraging results in mouse or ferret models, inconsistent results have been obtained in poultry (5, 9, 11, 12, 19–21).

A growing body of evidence emphasizes the importance of mucosal vaccination in the fight against IAV (15, 22–24). Current mucosal anti-IAV vaccines licensed in humans are intranasal cold-adapted live attenuated influenza vaccines (LAIV) displaying limited replication at high temperatures of the lower respiratory tract, but efficient replication at lower temperatures of the upper respiratory tract. Alternative attenuation strategies for the generation of LAIV under development in mammals and birds include the use of non-structural protein 1 (NS1) deficient, altered or truncated viruses, codon deoptimized viruses, or single-cycle infectious viruses obtained after mutation, deletion or substitution of viral components such as the transmembrane and cytoplasmic domains of M2 protein (5, 25, 26). LAIV have the potential to generate local and systemic humoral and cell-mediated immune responses and provide broad protection against IAV infections. However, some drawbacks have been associated with the use of LAIV such as safety issues related to the possible reversion of the vaccine strains into virulent strains by mutations or genetic reassortment with other circulating strains (27). Besides LAIV, mucosal inactivated subunit vaccines are being developed. To ensure the immuno-availability and the immuno-stimulating capacity of the IAV antigen(s), innovative delivery/adjuvant systems for subunit vaccines have been successfully developed in pre-clinical and clinical tests, including micro/nanosized particulate carriers associated or not with immunopotentiators such as agonists of pattern recognition receptors (PRRs) including Toll-like receptors (TLRs) (15). These formulations boost the magnitude, the duration and/or the breadth of the immune responses directed against the IAV antigen(s) (15). In this context, we previously fused three tandem repeats of M2e peptide to a ring nanoplatform based on recombinant nucleoprotein (N) of the respiratory syncytial virus (RSV), which self-assembled in homogenous rings of about 15 nm diameter when expressed in *Escherichia coli* (“N-3M2e nanorings”) (28). Mice immunized intranasally with these nanorings developed potent M2e-specific local and systemic humoral responses and were significantly protected against homologous IAV challenge (28). Recent studies have focused on the P97 protein of *Mycoplasma hyopneumoniae*, the etiological agent of porcine enzootic pneumonia, which plays a major role in the bacterial adhesion to the respiratory epithelium and stimulates the production of pro-inflammatory cytokines during infection (29). The fusion of the C-terminal end of P97 protein (P97c) to viral proteins boosted the anti-viral immune responses in mice (30), and the adjuvant effect of P97c may be mediated through its interaction with TLR-5 (29).

The goal of this study is to evaluate the immunogenicity and the protective potential of chimeric nanorings bearing 3M2e and LAH epitopes in fusion or not with P97c as universal mucosal anti-IAV vaccines in mice and chickens.

## Materials and Methods

### Plasmid Constructions

The M2e (GenBank accession number BAV59614.1, 1-24 aa) and LAH (GenBank accession number CAA24272.1, 420-474 aa) sequences derived from A/Puerto Rico/8/1934 (H1N1) (PR8) strain, the M2e (GenBank accession number AGY42150.1, 1-24 aa) and LAH (GenBank accession number AGY42146.1, 418-468 aa) sequences derived from A/mallard/Sweden/49/2002 (H5N9) strain, and the P97c sequence derived from *M. hyopneumoniae* 232 strain (GenBank accession #U50901.1, 799-1108 aa) were used in this study. The two cysteine residues of the M2e sequence (aa 17 and 19) were replaced by two serine residues to avoid formation of a disulfide bond that could perturb the assembly of nanorings (28). The pET-N-Sac plasmid was obtained from the pET-N plasmid, which contained the full-length coding sequence of N derived from RSV Long strain (ATCC VR-26, GenBank accession number AY911262.1), by introducing a SacI restriction enzyme site in frame and at the C-terminal end of the N sequence (28). The pET-N-3M2e plasmid containing three repetitions of the PR8 M2e sequence in frame and the C-terminal end of the N sequence was obtained previously (28). To obtain the pET-N-3M2e plasmid containing three repetitions of the H5N9 M2e sequence, the synthesized pUC57-3M2e plasmid (ProteoGenix, Schiltigheim, France) containing the H5N9 3M2e sequence was used as a template to amplify the 3M2e sequence by PCR using the Phusion high-fidelity DNA polymerase (Thermo Fisher Scientific, Waltham, MA, USA) with gene-specific primers flanked with SacI/SacI (forward primer/reverse primer) sites. The PCR-amplified 3M2e sequence was then digested by SacI enzyme and inserted into the pET-N-Sac plasmid using the same restriction site. The pET-LAH plasmid was obtained by inserting the PR8 LAH sequence between NdeI and XhoI sites into a pET-22 vector. The pET-N-LAH plasmid was obtained by inserting the PR8 or H5N9 LAH sequence containing a linker sequence GCCGGAGCA at its N-terminal end between the SacI and XhoI sites into the pET-N-Sac plasmid. The pET-N-P97c plasmid was obtained by inserting the P97c sequence containing a linker sequence GGCGGAAGC at its N-terminal end between the SacI and EcoRI sites into the pET-N-Sac plasmid. The pET-N-3M2e-P97c plasmid was obtained by inserting the PR8 3M2e sequence at the SacI site into the pET-N-P97c plasmid. The pET-N-P97c-3M2e plasmid was obtained by inserting the P97c sequence containing a linker sequence GGCGGAAGC at its N-terminal end at the SacI site into the pET-N-3M2e plasmid. The pET-N-LAH-P97c and pET-N-P97c-LAH plasmids were obtained by inserting the PR8 LAH and P97c sequences at the SacI site into the pET-N-P97c and pET-N-LAH plasmids, respectively. The LAH-P97c and P97c-LAH gene fragments were PCR-amplified using the pET-N-LAH-P97c and pET-N-P97c-LAH plasmids as templates and gene-specific primers flanked with NdeISacI/XhoI and NdeI/SacIXhoI sites, respectively. NdeI/XhoI-digested LAH-P97c and P97c-LAH PCR products were finally inserted into a pET-22 vector identically treated with restriction enzymes to generate the pET-LAH-P97c and pET-P97c-LAH plasmids, respectively. The PCR-amplified PR8 3M2e sequence flanked with SacI/SacI site was digested by SacI and inserted into either the SacI-digested pET-LAH-P97c plasmid to generate the pET-3M2e-P97c plasmid, or into the SacI-digested pET-P97c-LAH plasmid to generate the pET-P97c-3M2e plasmid. The primers and the restriction enzymes were purchased from Sigma-Aldrich (Saint-Louis, MO, USA) and Thermo Fisher Scientific, respectively. The entire coding sequences of all plasmid constructions were validated on both strands by DNA sequencing. All sequences and primers are available upon request.

### Expression and Purification of Proteins

The purification of nanorings depends upon specific interactions between the C-terminus of the phosphoprotein P of RSV (161-241 aa) (PCT) fused to glutathione *S*-transferase and the N protein, as previously described (31). Briefly, *E. coli* Rosetta 2 (DE3) competent cells (Novagen, Madison, WI, USA) co-transformed with the pGEX-PCT plasmid and either the pET-N-Sac, pET-N-P97c, pET-N-LAH, pET-N-3M2e, pET-N-LAH-P97c, pET-N-P97c-LAH, pET-N-3M2e-P97c or pET-N-P97c-3M2e plasmid were grown at 37°C for 8 h in Luria-Bertani (LB) medium containing 100 µg/ml ampicillin, 50 µg/ml kanamycin and 40 µg/ml chloramphenicol. The protein expression was induced by adding 0.8 mM isopropyl-β-D-thio-galactoside (IPTG) to the medium and bacteria were incubated overnight at 28°C. Bacterial pellets were then incubated for 1 h on ice in lysis buffer (50 mM Tris-HCl pH 8, 60 mM NaCl, 1 mM EDTA, 2 mM dithiothreitol (DTT), 0.2% Triton X-100, 1 mg/ml lysozyme) supplemented with complete protease inhibitor cocktail (Roche, Basel, Switzerland), then sonicated, and finally centrifuged at 4°C for 30 min at 10,000 × *g*. Supernatants were incubated overnight with glutathione-sepharose 4B beads (GE Healthcare, Chicago, IL, USA). Beads were then washed three times in lysis buffer and three times in phosphate-buffer saline (PBS), and incubated with thrombin (Novagen) overnight at 20°C. Recombinant nanorings separated from the beads were finally loaded onto a HiLoad Superdex 200 column (GE Healthcare) using 20 mM Tris-HCl (pH 8.5) and 150 mM NaCl as the eluent.

The purification of LAH, LAH-P97c, P97c-LAH, 3M2e-P97c or P97c-3M2e relied on the presence of a 6xHis-tag located at the C-terminal end of the coding sequence. *E. coli* competent cells transformed with the pET-LAH, pET-LAH-P97c, pET-P97c-LAH, pET-3M2e-P97c or pET-P97c-3M2e plasmid were grown at 37°C for 8 h in LB medium containing 100 µg/ml ampicillin and 40 µg/ml chloramphenicol. After protein expression, bacterial pellets were re-suspended in lysis buffer (20 mM Tris-HCl pH 8, 500 mM NaCl, 0.1% Triton X-100, 10 mM imidazole, 1 mg/ml lysozyme) supplemented with complete protease inhibitor cocktail, incubated for 1 h on ice, sonicated, and then centrifuged at 4°C for 30 min at 10,000 × *g*. Supernatants were then loaded onto a HiTrap immobilized metal ion affinity chromatography (IMAC) column (GE Healthcare) charged with 0.2 M NiSO_4_. The column was washed in the washing buffer (20 mM Tris-HCl pH 8.5, 500 mM NaCl, 50 mM imidazole) and proteins were eluted in the same buffer with 500 mM imidazole before being loaded onto a HiLoad Superdex 200 column using 20 mM Tris-HCl (pH 8.5) and 150 mM NaCl as the eluent. P97c and 3M2e peptides were synthesized by ProteoGenix. The absence of endotoxin was tested by a Limulus amebocyte lysate (LAL) endotoxin quantification kit (Thermo Fisher Scientific) with a sensitivity limit of 0.1 endotoxin unit/ml.

### SDS-PAGE and Western Blot Assays

Proteins were prepared in Laemmli buffer (62.5 mM Tris-HCl pH 6.8, 5% β-mercaptoethanol, 2% sodium dodecyl sulfate (SDS), 20% glycerol, 0.01% bromophenol blue) and denatured 5 min at 95°C. Samples were then run on 12.5% SDS-polyacrylamide gels with to ProSieve Prestained protein ladder and detected by Coomassie brilliant blue staining. Alternatively, gels were electroblotted onto a nitrocellulose membrane for 1 h at 20 V. Membranes were blocked with PBS containing 5% skim milk during 1 h at room temperature (RT) and then incubated overnight at 4°C with either a rabbit anti-N polyclonal sera (Pab) (1:5,000) (32), a mouse anti-M2 monoclonal antibody (Mab) (1:5,000; clone 14C2) (Santa Cruz Biotechnology, Dallas, TX, USA) or a mouse anti-P97c Mab (1:3,000; clone 8H4-G6) (30) in PBS with 0.3% Tween 20 (PBS-T 0.3%) and 5% skim milk. Membranes were washed in PBS-T 0.3% and finally incubated 1 h at RT with relevant peroxidase-conjugated secondary antibodies (SeraCare, Milford, MA, USA) diluted in blocking solution. Proteins were visualized by chemiluminescence with Clarity Western ECL substrate (Bio-Rad, Hercules, CA, USA).

### Size Measurements of Nanorings by Dynamic Light Scattering (DLS)

Size measurements with the Zetasizer Nano series (Malvern Panalytical, Malvern, UK) based on the principle of DLS, were made at 20°C using a helium-neon laser wavelength of 633 nm and detection angle of 173°. The results were presented as size distribution by volume calculated from the Malvern software (28).

### Visualization of Nanorings by Transmission Electron Microscope (TEM)

Electron micrographs were acquired using a CM12 TEM (Royal Philips Electronics, Amsterdam, Netherlands) at 80 kV excitation voltage. Samples at 0.05-1 mg/ml were applied onto an airglow- discharged carbon-coated 200-mesh copper grid and stained with a 2% uranyl acetate aqueous solution (28).

### *In Vivo* Experiments in Mice

Six to 7-week-old female BALB/c mice were purchased from Janvier Labs (Le Genest-Saint-Isle, France) and housed under specific-pathogen-free (SPF) conditions in a biosafety level 2 facility (IERP, INRAE, Jouy-en-Josas, France) with access to food and water *ad libitum*. All mouse experiments were carried out in accordance with INRAE guidelines, which are compliant with the European animal welfare regulation. The protocols were approved by the Animal Care and Use Committee at the Centre de Recherche de Jouy-en-Josas (COMETHEA) under relevant institutional authorization (Ministère de l’éducation nationale, de l’enseignement supérieur et de la recherche; authorization number 2015100910396112v1, APAFIS number 1487).

Three sets of experiments were conducted to evaluate the immunogenicity of vaccine candidates bearing PR8 epitopes and the immunostimulatory effect of nanorings and P97c. In the first set of experiments, mice were anesthetized with a solution of ketamine and xylazine (50 and 10 mg/kg of body weight, respectively) and immunized three times, at 2-week intervals, by intranasal instillation of 50 µl of endotoxin-free PBS containing 20 µg of nanorings bearing PR8 epitopes in fusion or not to P97c (N-LAH, [*n* = 10]; N-3M2e, [*n* = 8]; N-LAH-P97c, [*n* = 9]; N-P97c-LAH, [*n* = 9]; N-3M2e-P97c, [*n* = 9]). Control groups received intranasal administrations of 20 µg of free PR8 peptides (LAH, [*n* = 8]; 3M2e, [*n* = 7]) or PR8 peptides fused to P97c (LAH-P97c, [*n* = 9]; P97c-LAH, [*n* = 9]; 3M2e-P97c, [*n* = 10]; P97c-3M2e, [*n* = 9]). Preparations which did not contain P97c were adjuvanted with 5% MG (SEPPIC, Air Liquide, La Garenne-Colombes, France). Negative control groups received preparations without PR8 epitopes (P97c (20 µg), [*n* = 5]; N (20 µg) + MG, [*n* = 8]; N-P97c (20 µg), [*n* = 10]; MG, [*n* = 5]). The antigen dose was selected according to previous studies (28). On day 42, sera were isolated from blood samples collected *via* cheek puncture. Mice were then sacrificed and bronchoalveolar lavage fluids (BAL) were collected by flushing the lungs *via* tracheal puncture with 1 ml PBS and clarified by centrifugation. In the second set of experiments, mice were immunized three times, at 2-week intervals, by intranasal instillation of 50 µl of endotoxin-free PBS containing nanorings bearing PR8 epitopes (N-LAH (20 µg) + MG, [*n* = 5]; N-3M2e (20 µg) + MG, [*n* = 5]). Control groups received intranasal administrations of free PR8 peptides (LAH (20 µg) + MG, [*n* = 5]; 3M2e (20 µg) + MG, [*n* = 5]) or subcutaneous injections of an equivalent of 100 lethal dose 50 (LD_50_) of UV-inactivated PR8 strain (iPR8, [*n* = 4]) admixed with MG. This latter group has been included in the study as an experimental control representative of current anti-IAV vaccines which are mainly inactivated viruses administered *via* the parenteral route (15). Negative control group received MG (*n* = 4). On day 42, spleens were collected to evaluate LAH- or M2e-specific cellular responses. In the last set of experiments, mice were immunized three times, at 2-week intervals, by intranasal instillation of 50 µl of endotoxin-free PBS containing nanorings bearing PR8 administered alone (N-LAH (20 µg) + MG, [*n* = 7]; N-3M2e (20 µg) + MG, [*n* = 7]), or in combination (N-3M2e (20 µg) + N-LAH (20 µg) + MG, [*n* = 8]). Control groups received intranasal administrations of free PR8 peptides (LAH (20 µg) + MG, [*n* = 7]; 3M2e (20 µg) + MG, [*n* = 7]) or MG (*n* = 4), or subcutaneous injections of [iPR8 + MG] (*n* = 7). On day 42, spleens (*n* = 4 for MG and [iPR8 + MG] groups; *n* = 5 for [N-LAH + MG] and [N-3M2e + MG] groups), sera and BAL were collected.

A final set of experiments aimed at evaluating the protective potential of nanorings bearing PR8 epitopes against an experimental homologous IAV infection. In a first stage, mice received three administrations of vaccine formulations or vehicle as described above (N-LAH + MG, [*n* = 10]; N-3M2e + MG, [*n* = 10]; N-3M2e + N-LAH + MG, [*n* = 10]; LAH + MG, [*n* = 10]; 3M2e + MG, [*n* = 10]; MG, [*n* = 9]). Two weeks after the last immunization, mice were anesthetized and inoculated intranasally with 15 LD_50_ of PR8 strain in 50 µl endotoxin-free PBS. Mice immunized with [iPR8 + MG] (*n* = 8) were included as a positive control group because conventional vaccination with inactivated virus is known to confer protection against homologous challenge with the induction of neutralizing anti-HA antibodies contributing critically to viral clearance (15, 28). Body weight and mortality of each mouse were monitored daily until 14 days post-infection (p.i.). Mice that had lost 20% or more of their initial weight were euthanized according to ethical endpoints. In a second stage, mice received three administrations of vaccine formulations or vehicle and were then infected with 15 LD_50_ of PR8 strain as described above. Four days after infection, individual viral loads were measured from lung homogenates (N-LAH + MG, [*n* = 4]; N-3M2e + MG, [*n* = 5]; N-LAH + N-3M2e + MG, [*n* = 4]; LAH + MG, [*n* = 5]; 3M2e + MG, [*n* = 5]; iPR8 + MG, [*n* = 4]; MG, [*n* = 5]) by quantitative real-time polymerase chain reaction (qRT-PCR).

### *In Vivo* Experiments in Chickens

Experimentations were conducted in accordance with the European Council Directive CEE86/609 and animal protocols approved by the Ethics Committee “Sciences et santé animale”, committee number 115. Three-week-old white Leghorn chickens were purchased from the Plateforme d’Infectiologie Expérimentale (INRAE, Nouzilly, France) and housed under SPF conditions in a biosafety level 2 facility at the National Veterinary School of Toulouse (France) (experimental unit agreement number C3155527) with access to food and water *ad libitum*. Birds were transferred in biosafety level 3 cabinets (I-Box; Noroit, Nantes, France) under negative pressure with HEPA-filtered air before experimental AIV infection. Chickens were immunized three times at 2-week intervals with a combination of two chimeric nanorings bearing H5N9 LAH and 3M2e epitopes. The first group received 100 µl of endotoxin-free PBS containing 50 µg of each chimeric nanorings adjuvanted with 70% Montanide™ ISA 71 VG (ISA) (SEPPIC) intramuscularly (N-LAH + N-3M2e + ISA, [*n* = 15]). ISA is a blend of oil and an ester from mannitol sugar and oleic fatty acid (anhydromannitol octadecenoate ether) with specific emulsifying properties due to its sugar polar head, its non-ionicity and the specificity of fatty acid chains of the surfactant system, which is extensively used in parenteral vaccination in chickens. The second group received 100 µl of endotoxin-free PBS containing 25 µg of each chimeric nanorings adjuvanted with MG by choanal route in addition to 40 µl of endotoxin-free PBS containing 25 µg of each chimeric nanorings adjuvanted with MG by eye drop (20 µl/eye) (N-LAH + N-3M2e + MG, [*n* = 15]). Non-vaccinated control group received PBS (*n* = 16). On day 46, sera were isolated from blood samples collected *via* jugular vein punction and tears were obtained by sprinkling salt in an eye of each bird (*n* = 3-5 per group) and collecting the fluid with a micropipette (33). On day 49, chickens were inoculated *via* the choanal route with 5 LD_50_ of HPAIV A/duck/Tarn/RG/2016 (H5N8) strain in 100 µl endotoxin-free PBS and the mortality was recorded daily until 7 days p.i.

### Antigen-Specific Antibody Titration by ELISA

Microtiter plates (Immulon 2HB, Thermo Fisher Scientific) were coated overnight at 4°C with synthesized LAH or M2e (ProteoGenix) derived from PR8 (for the analysis of mouse samples) or H5N9 (for the analysis of chicken samples) strains (200 ng per well in 100 µl carbonate-bicarbonate buffer 0.1 M pH 9.5). Plates were washed five times with PBS-T 0.05% between each step of the assay. After coating, the plates were blocked with PBS-T 0.05% and 5% skim milk for 2 h at RT. Sera, BAL and LS were serially diluted (2-fold dilutions) in PBS-T 0.05% and 5% skim milk (starting at 1:3 for LS and at 1:50 for serum/BAL) and incubated for 1 h at RT. Negative wells contained only PBS-T 0.05% and 5% skim milk. Antigen-bound mouse antibodies were detected using peroxidase-conjugated goat anti-mouse IgG (H+L) (1:5,000) (KPL, Gaithersburg, MD, USA), IgA (1:5,000) (Southern Biotech, Birmingham, AL, USA), IgG2b (1:1,000) (Southern Biotech), or IgG3 (1:1,000) (Southern Biotech), or peroxidase-conjugated rat anti-mouse IgG1 (1:1,000) (BD Biosciences, Franklin Lakes, NJ, USA) or IgG2a (1:1,000) (BD Biosciences). Antigen-bound chicken antibodies were detected using peroxidase-conjugated goat anti-chicken IgG (Fc) (1:50,000 or 1:10,000 for sera or LS, respectively) (Bio-Rad) or IgA (1:10,000) (Bio-Rad). After incubation for 1 h at RT, plates were developed with 3,3’,5,5’-tetramethylbenzidine (TMB) substrate (KPL), and the enzyme reaction was stopped by addition of 1 M H_2_SO_4_. Absorbance was read at 450 nm with an Infinite 200 Pro microplate reader (Tecan, Männedorf, Switzerland). The reciprocal of the last sample dilution that resulted in an optical density at 450 nm (OD_450_) ≤ twice the OD_450_ of negative wells (cutoff) was considered the titer of that sample. When the OD_450_ of the first dilution of a sample was lower than the cutoff, its titer was arbitrarily fixed to 3 or 50 for LS or sera/BAL, respectively. Optimal dilutions of the coating antigen (LAH or M2e) and peroxidase-conjugated anti-mouse or anti-chicken antibodies were determined during preliminary standardizations.

### Preparation of Mouse Spleen Cells

Individual spleens were mechanically disrupted in sterile RPMI-1640 medium (Thermo Fisher Scientific) supplemented with 10% heat-inactivated fetal calf serum (FCS, Eurobio Scientific, Les Ulis, France) and filtered through a 100 µm nylon filter. After incubation with NH_4_Cl lysing buffer (Sigma-Aldrich) to remove red blood cells, total spleen cells were adjusted to 5 x 10^6^ viable cells/ml in complete medium consisting of RPMI-1640 medium supplemented with 10% FCS, 2 mM L-glutamine, 50 µM β-mercaptoethanol and 100 U/ml penicillin-streptomycin (Thermo Fisher Scientific) and were re-stimulated *ex vivo* to evaluate specific cellular immune responses by ELISA and ELISpot assay (see below). All solutions were tested for the absence of endotoxin by the LAL test. Any possible residual endotoxin during cell stimulation was controlled by the addition of polymyxin B sulfate (MilliporeSigma, Burlington, MA, USA) at 20 µg/ml (34).

### Cytokine Quantification by ELISA

Mouse spleen cells were distributed in 96-well flat-bottom cell culture plates (5 × 10^5^ cells/well) and incubated in triplicate with synthesized LAH or M2e (2 µg/well) as activators or with complete medium as negative control. Cell cultures were incubated at 37°C in 5% CO_2_ for 72 h, and supernatants were harvested. Levels of interferon gamma (IFN-γ), interleukin-17A (IL-17A), IL-4, IL-5, IL-6 and IL-10 were measured from supernatants by sandwich ELISA using pair-matched antibodies from eBioscience (San Diego, CA, USA) (IFN-γ, IL-4, IL-5, IL-6, IL-10) or BD Biosciences (IL-17A). Two-fold dilutions of recombinant mouse cytokines were used to generate standard curves. Sample dilutions giving OD_450_ readings in the linear portion of the appropriate standard curve were used to quantify the levels of each cytokine.

### ELISpot Assay

Ninety-six-well MultiScreen_HTS_-IP polyvinylidene fluoride membrane plates (MilliporeSigma) were coated overnight at 4°C with capture anti-mouse IFN-γ Mab (clone R4-6A2, BD Biosciences) or IL-17A Mab (clone TC11-18H10) (1 µg per well in 100 µl PBS). Plates were then washed three times with sterile PBS and blocked with complete medium for 2 h at 37°C. Mouse spleen cells were serially diluted (two-fold dilution) in complete medium (starting at a concentration of 5 × 10^6^ cells/ml) and 100 µl/well of each dilution was incubated with synthesized LAH or M2e (2 µg/well) for 24 h at 37°C.

Negative control wells were coated wells containing complete medium or uncoated wells containing 5 × 10^5^ cells. Subsequently, plates were washed in PBS-T 0.05% and incubated with 100 µl/well of biotinylated rat anti-mouse IFN-γ Mab (clone XMG1.2) or IL-17A Mab (clone TC11-8H4) at 2 µg/ml in PBS-T 0.05% supplemented with 1% BSA for 2 h at 37°C. After further washes and 45 min incubation with streptavidin-alkaline phosphatase at 1 µg/ml (Mabtech, Nacka Strand, Sweden), IFN-γ- or IL-17A- secreting cells were visualized by adding BCIP-NBT (5-bromo-4-chloro-3-indolyl-phosphate/nitro blue tetrazolium; Thermo Fisher Scientific) substrate for 30 min. The spots were counted using the iSPOT reader from AID Autoimmun Diagnostica GmbH (Straßberg, Germany). The background from the negative wells was subtracted and the results were expressed as the number of spot-forming cells per 5 × 10^5^ input cells. Optimal dilutions of coating antibodies and biotinylated anti-mouse antibodies were determined during preliminary standardizations.

### Determination of Pulmonary Viral Loads by qRT-PCR

Total RNA was extracted from mouse lung homogenates using the RNeasy Plus Mini Kit (Qiagen, Venlo, Netherlands) and 100 ng of RNA samples were reverse-transcribed with SuperScript II Reverse Transcriptase (Thermo Fisher Scientific) using the specific IAV M1 primer: 5’-TCT AAC CGA GGT CGA AAC GTA-3’ (35). Resulting cDNA samples were mixed with iTaq universal SybR green PCR supermix (Bio-Rad) and primers targeting a conserved region of the PR8 M1 gene (sense: 5’-TCT AAC CGA GGT CGA AAC GTA-3’; antisense: 5’-AGG GCA TTT TGG ACA AAG CGT CTA-3’). The qRT-PCR program was run on the MasterCycler R realplex (Eppendorf, Montesson, France) as follows: an initial DNA denaturation step at 95°C for 3 min, then 40 cycles composed firstly of a denaturation step at 95°C for 15 s, then an annealing step at 64°C for 15 s, and finally an extension step at 72°C for 30 s. Each cDNA sample and non-template controls were run in triplicate. To ensure that primers produced a single and specific PCR amplification product, a dissociation curve was performed at the end of the PCR cycle. In each assay, serial ten-fold dilutions of the pPOLI-M/PR8 plasmid (Pr. Ervin Fodor, University of Oxford, UK) were run in duplicate, allowing to quantify the number of M1 gene copies generated from unknown samples by comparison of the cycle threshold values using the Realplex software (Eppendorf). Results were expressed as the number of copies of M1 RNA per 100 ng of input total lung RNA.

### Statistical Data Analysis

The log-rank (Mantel-Cox) test was used to compare the survival rates between the mouse groups. Otherwise, differences between the experimental groups were analyzed for significance using the Mann-Whitney rank sum test. All analyses were done using the Sigma Plot system v11.0 (Systat Software, San Jose, CA, USA). A *P* value < 0.05 was considered statistically significant.

## Results

### Biochemical and Biophysical Characterization of LAH- and M2e-Based Fusion Proteins

**Figure 1:**
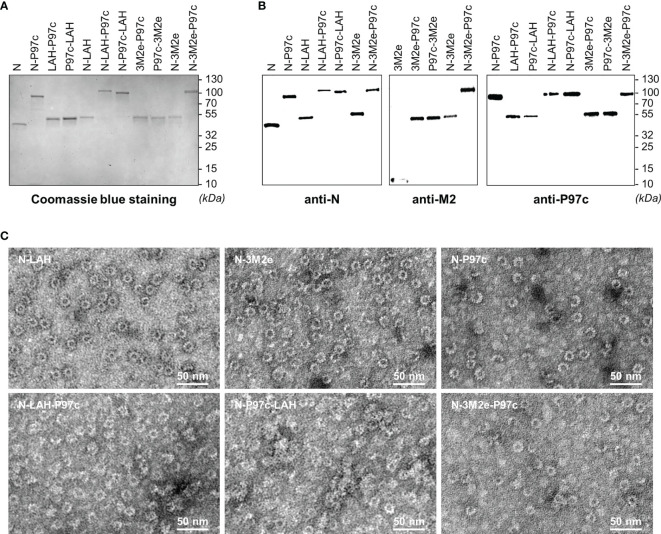
Biochemical and biophysical characterization of LAH- and 3M2e-based fusion proteins. Recombinant nanorings bearing PR8 epitopes in fusion or not with P97c (N-LAH, N-3M2e, N-LAH-P97c, N-P97c-LAH, N-3M2e-P97c), fusion proteins composed of PR8 epitopes and P97c (LAH-P97c, P97c-LAH, 3M2e-P97c, P97c-3M2e), nanorings bearing P97c (N-P97c) and naked nanorings (N) were produced and purified as described in “Materials and Methods” and then analyzed by **(A)** SDS-PAGE followed by Coomassie blue staining and **(B)** Western blot assay with (**B**, left panel) N-specific Pab, (**B**, central panel) M2-specific Mab and (**B**, right panel) P97c-specific Mab. **(C)** Nanorings were negatively stained and observed by transmission electron microscopy.

In this study, we explored the adjuvant potential of N nanorings and P97c for the development of efficient anti-IAV vaccine formulations containing conserved IAV epitopes. To this end, we produced a series of chimeric nanorings with PR8 (H1N1) epitopes linked alone or in combination with P97c at the C-terminal end of the N sequence exposed at the surface of nanorings (36). Different combinations of PR8 epitopes and P97c were conceived because the fusion protein orientation could influence the characteristics of the humoral response generated against the viral epitopes (30). M2e-based constructions were designed with three M2e copies to increase the immunogenicity of the peptide (28). Five different chimeric nanorings were thus created, namely N-LAH, N-3M2e, N-LAH-P97c, N-P97c-LAH and N-3M2e-P97c. In parallel, we produced LAH-P97c, P97c-LAH, 3M2e-P97c and P97c-3M2e fusion proteins. Naked nanorings (N) and nanorings bearing P97c (N-P97c) were generated as controls. Due to very low production yield, N-P97c-3M2e nanorings were excluded from the study. Analysis by SDS-PAGE followed by Coomassie blue staining revealed the presence of a unique band at the expected theorical molecular weight for each sample **(** **Figure 1A** **)**. Western blot assays using specific antibodies confirmed the identity and antigenicity of each preparation **(** **Figure 1B** **)**. The presence of LAH epitopes was validated by liquid chromatography coupled with tandem mass spectrometry (data not shown). Observation by TEM showed that nanorings carrying IAV and/or P97c epitopes formed similar ring-like structures as those previously observed with N or N-3M2e nanorings (28) **(** **Figure 1C** **)**. DLS analysis indicated that N and N-3M2e nanoring preparations were mainly composed of a homogenous population with a hydrodynamic radius of 17 nm and 18 nm, respectively, in accordance with previous observations (28, 31). A similar DLS profile with a hydrodynamic radius of 18 nm was obtained with the N-LAH nanoring preparation **(** **Supplemental Figure 1****)**. The size of the particles was increased after the fusion of P97c to nanorings, and a major population with a hydrodynamic radius of 25 nm, 41 nm, 32 nm and 34 nm was detected with N-P97c, N-LAH-P97c, N-P97c-LAH and N-3M2e-P97c samples, respectively. Therefore, nanorings are versatile nanoplatforms on which different IAV epitopes can be grafted alone or in combination with adjuvant sequence (P97c) without affecting the self-assembly and the structural integrity of nanorings.

### Evaluation of the Immunopotentiator Effect of Nanorings and P97c on LAH- and M2e-Specific Systemic and Mucosal Humoral Responses in Mice

**Figure 2:**
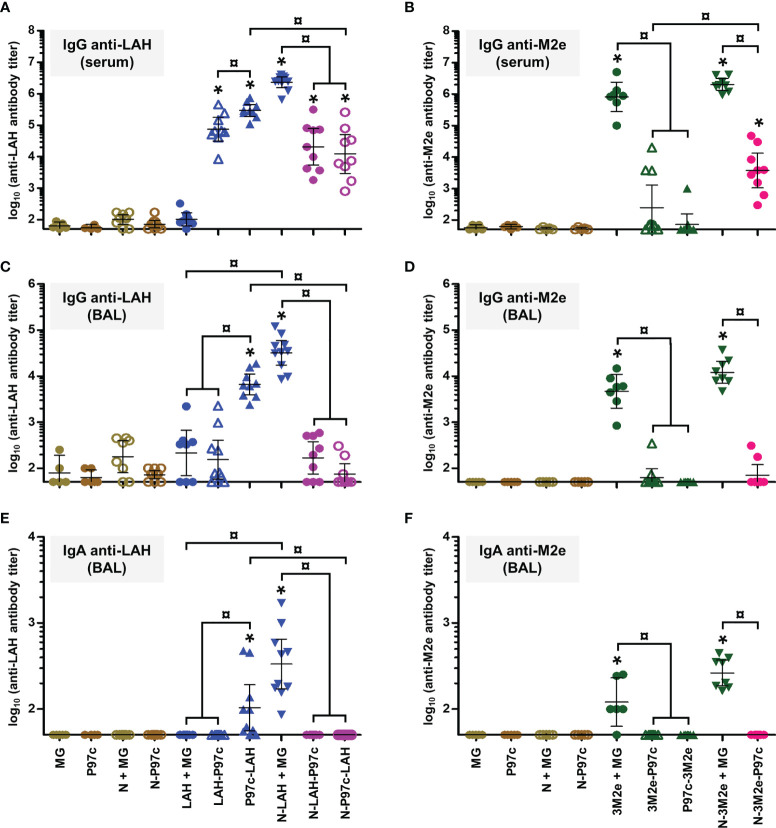
Evaluation of the immunopotentiator effect of nanorings and P97c on LAH- and M2e-specific systemic and mucosal humoral responses in mice. Mice received at 2-week intervals three intranasal administrations of LAH or 3M2e peptides fused to nanorings and/or P97c or, as controls, free LAH or 3M2e peptides. Preparations without P97c were admixed with Montanide™ gel (MG). Mice receiving P97c nanorings (N-P97c), naked nanorings (N + MG), P97c or MG were included as negative control groups. Two weeks after the third immunization, **(A)** anti-LAH or **(B)** anti-M2e IgG titers in the serum, and anti-LAH **(C)** IgG and **(E)** IgA or anti-M2e **(D)** IgG and **(F)** IgA titers in the bronchoalveolar lavage fluids (BAL) were determined by ELISA. The titer of each mouse sample is presented, including the geometric mean with 95% confidence interval. *, statistically significant difference (*P <* 0.05) in comparison to the respective negative control groups (MG, P97c, N + MG, N-P97c). ¤, statistically significant difference (*P <* 0.05) between the indicated groups.

In a first set of experiments, we evaluated the immunopotentiator effect of nanorings and P97c on the development of humoral immunity directed against IAV epitopes in mice. Animals received three intranasal administrations of LAH or 3M2e peptides derived from PR8 strain fused to nanorings and/or P97c. Vaccine preparations which did not contain P97c were admixed with MG, a commercial adjuvant efficient in mucosal immunization (28). The magnitude and composition of systemic and local antibody responses directed against LAH or M2e were evaluated **(** **Figure 2** **)**.

Concerning LAH-specific humoral responses, mice vaccinated with [LAH + MG] did not present significant systemic **(** **Figure 2A** **)** or mucosal **(** **Figures 2C, E** **)** antibody titers. In contrast, mice immunized with P97c-LAH or LAH-P97c fusion proteins developed a potent systemic IgG response, and the antibody titers were significantly higher in the P97c-LAH group than in the LAH-P97c group **(** **Figure 2A** **)**. Nonetheless, although IgG were detected in the BAL of the P97c-LAH group, overall mice presented low to negligible IgA titers, and the mucosal humoral response of [LAH-P97c]-instilled mice was not significantly different from negative control groups **(** **Figures 2C, E** **)**. The LAH-specific humoral response of mice immunized with N-LAH-P97c or N-P97c-LAH nanorings was only composed of serum IgG, whose titers were not increased in comparison to mice immunized with LAH-P97c or P97c-LAH fusion proteins, respectively, and no significant mucosal antibody titers could be measured **(** **Figures 2C, E** **)**. Finally, the [N-LAH + MG] formulation triggered the strongest humoral responses, composed of serum IgG as well as local IgG and IgA.

Regarding M2e-specific humoral responses, mice vaccinated with [3M2e + MG] exhibited significant systemic IgG titers **(** **Figure 2B** **)** and local IgG **(** **Figure 2D** **)** and IgA **(** **Figure 2F** **)** titers in contrast with [3M2e-P97c]- or [P97c-3M2e]-vaccinated mice. There was a tendency towards higher levels of antibodies in serum and BAL in the [N-3M2e + MG] group compared to the [3M2e + MG] group, however these differences were not statistically significant **(** **Figures 2B, D, F** **)**. N-3M2e-P97c nanorings induced modest serum IgG titers **(** **Figure 2B** **)** but no mucosal Ig were detected **(** **Figures 2D, F** **)**.

To summarize, while fusion of P97c to LAH boosted LAH-specific systemic humoral responses, no adjuvant effect of P97c could be observed towards 3M2e. The fusion of LAH to nanorings boosted both systemic and local LAH-specific antibody responses, whereas the carrier effect of nanorings was less pronounced towards 3M2e. Chimeric proteins composed of the IAV epitopes fused to P97c either administered alone or in fusion with nanorings were unable to trigger potent mucosal humoral responses and were thus excluded from the following experiments. In contrast, preparations incorporating N-LAH or N-3M2e nanorings admixed with MG were shown to be efficient mucosal vaccine formulations.

### Evaluation of the Immunopotentiator Effect of Nanorings on LAH- and 3M2e-Specific Cell-Mediated Immune Responses in Mice

Both humoral and cellular immune defenses are involved in the fight against IAV infections. Accordingly, we analyzed the characteristics of LAH- and M2e-specific cell-mediated immunity generated in [N-LAH + MG]- and [N-3M2e + MG]-vaccinated mice, respectively. The carrier effect of nanorings was investigated and the cellular response of mice immunized with the inactivated homologous IAV strain (iPR8) *via* the parenteral route was analyzed in parallel. We focused our study on prototypical type 1 (IFN-γ), type 2 (IL-4, IL-5 and IL-10), and type 17 (IL-17A) cytokines and on IL-6, a pleiotropic cytokine with multiple immunoregulatory effects including the promotion of T helper (Th) 2 and Th17 responses as well as antibody responses and mucosal IgA immunity (37).

**Figure 3:**
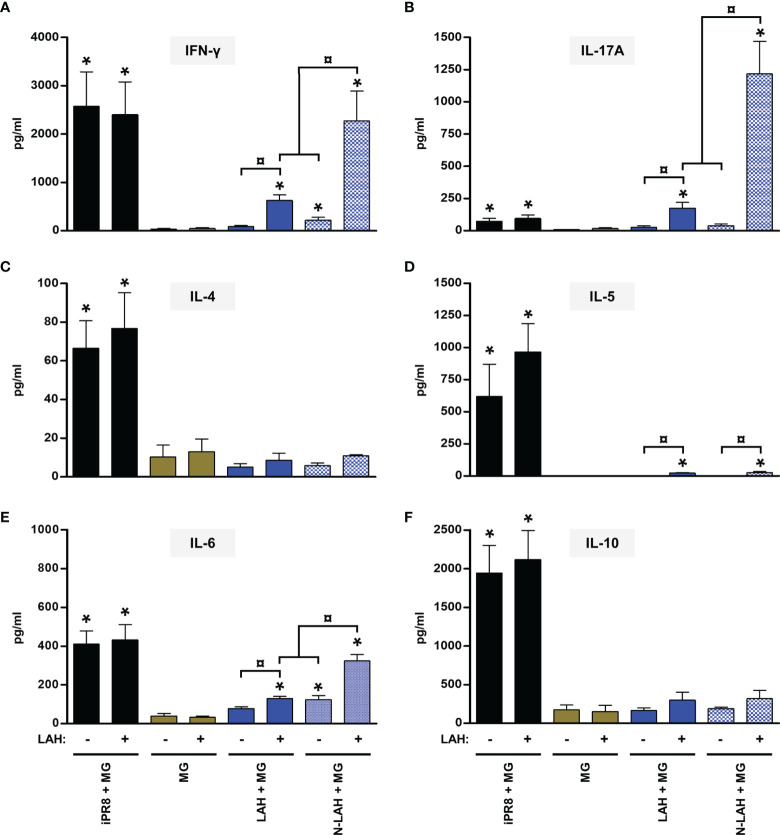
Evaluation of the immunopotentiator effect of nanorings on LAH-specific cellular responses in mice. Mice received at 2-week intervals three intranasal administrations of LAH peptide fused to nanorings admixed with Montanide™ gel (MG). Experimental control groups received intranasal administrations of free LAH peptide admixed with MG or subcutaneous administrations of UV-inactivated PR8 strain (iPR8) admixed with MG. Vaccine vehicle (MG) was intranasally administered to the negative control group. Two weeks after the third immunization, spleen cells from individual mice were restimulated *ex vivo* for 72 h in presence (‘+’) or absence (‘-’) of synthesized LAH peptide. The levels of secreted **(A)** IFN-γ, **(B)** IL-17A, **(C)** IL-4, **(D)** IL-5, **(E)** IL-6 and **(F)** IL-10 in the culture supernatants were quantified by ELISA. Data are presented as arithmetic means with SEM of 4-5 individual spleens. *, statistically significant difference (*P <* 0.05) in comparison to MG group. ¤, statistically significant difference (*P <* 0.05) between the indicated groups.

**Figure 4:**
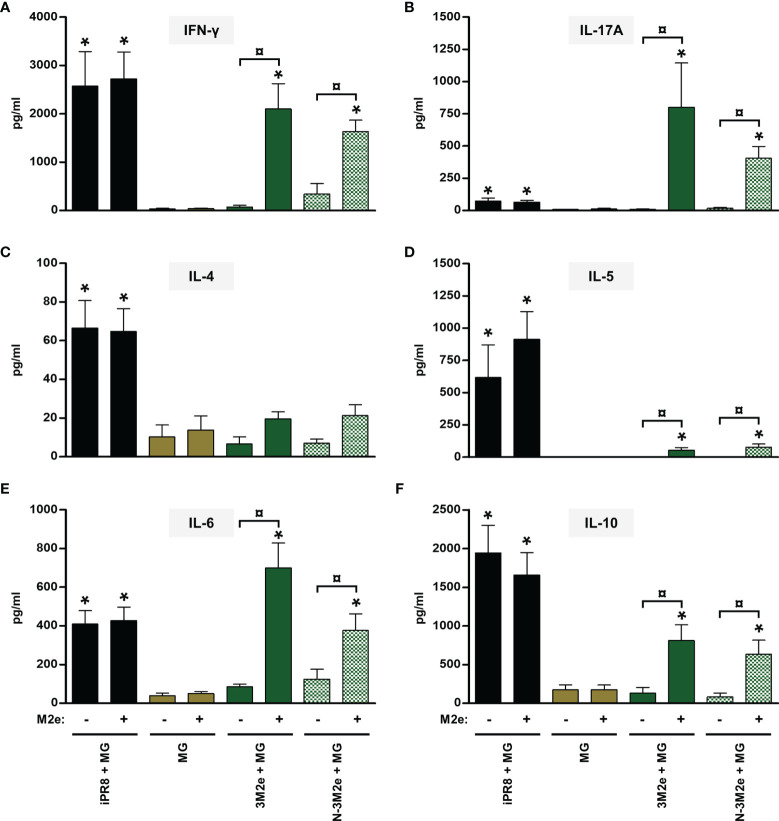
Evaluation of the immunopotentiator effect of nanorings on M2e-specific cellular responses in mice. Mice received at 2-week intervals three intranasal administrations of 3M2e peptide fused to nanorings admixed with Montanide™ gel (MG). Experimental control groups received intranasal administrations of free 3M2e peptide admixed with MG or subcutaneous administrations of UV-inactivated PR8 strain (iPR8) admixed with MG. Vaccine vehicle (MG) was intranasally administered to negative control group. Two weeks after the third immunization, spleen cells from individual mice were restimulated *ex vivo* for 72 h in presence (‘+’) or absence (‘-’) of synthesized M2e peptide. The levels of secreted **(A)** IFN-γ, **(B)** IL-17A, **(C)** IL-4, **(D)** IL-5, **(E)** IL-6 and **(F)** IL-10 in the culture supernatants were quantified by ELISA. Data are presented as arithmetic means with SEM of 4-5 individual spleens. *, statistically significant difference (*P <* 0.05) in comparison to MG group. ¤, statistically significant difference (*P <* 0.05) between the indicated groups.

Mice vaccinated with [LAH + MG] developed LAH-specific cellular responses with type 1 and type 17 cytokine profiles as seen by the detection of IFN-γ **(** **Figure 3A** **)** and IL-17A **(** **Figure 3B** **)** in culture supernatants of LAH-restimulated spleen cells, respectively. Low levels of IL-5 **(** **Figure 3D** **)** and IL-6 (**Figure 3E** **)** were also measured, whereas there were no differences in the levels of IL-4 or IL-10 between LAH-restimulated cells isolated from [LAH + MG]- and MG-instilled mice **(** **Figures 3C, F** **)**. The cell-mediated immune response of mice vaccinated with [N-LAH + MG] exhibited similar cytokine profiles, with higher levels of secreted IFN-γ, IL-6 and IL-17A than [LAH + MG]-immunized mice. Mice vaccinated with [3M2e + MG] developed M2e-specific cellular responses with type 1 **(** **Figure 4A** **)** and type 17 **(** **Figure 4B** **)** cytokine profiles. While M2e-restimulated cells isolated from [3M2e + MG]-vaccinated mice secreted significant levels of IL-10 **(** **Figure 4F** **)** and IL-6 **(** **Figure 4E** **)**, the cells released low levels of IL-5 **(** **Figure 4D** **)** and no significant amount of IL-4 could be detected **(** **Figure 4C** **)**. The M2e-specific cellular responses were generally similar in [3M2e + MG] and [N-3M2e + MG] groups. High levels of IFN-γ, IL-4, IL-5, IL-6, and IL-10 and low levels of IL-17A were detected in culture supernatants of spleen cells isolated from [iPR8 + MG]-vaccinated mice **(** **Figures 3**, **4** **)**. However, no differences were obtained between cells restimulated with PR8 peptides and non-restimulated cells. Thus, no cell-mediated immunity specifically directed against LAH or M2e epitopes could be measured in the spleen of mice immunized with [iPR8 + MG]. The quantification of LAH- **(** **Supplemental Figure 2A** **)** or M2e- **(** **Supplemental Figure 2B** **)** specific IFN-γ-secreting cells by ELISpot assay led to the same conclusions as those obtained with ELISA. An immunostimulatory effect of nanorings on 3M2e was nevertheless observed in ELISpot assay, which reported a higher frequency of M2e-specific IFN-γ-secreting cells in the [N-3M2e + MG] group than in the [3M2e + MG] group **(** **Supplemental Figure 2B** **)**.

Thus, administration of [N-LAH + MG] and [N-3M2e + MG] promoted the development of LAH- and M2e-specific cellular responses in mice, respectively, which were dominated by type 1 and type 17 cytokine profiles. In contrast, the inactivated homologous IAV strain was unable to trigger specific responses against the two conserved IAV epitopes. Nanorings exerted a potent immunostimulatory effect on the formation of LAH-specific cellular immunity, whereas the carrier effect of nanorings was less pronounced towards 3M2e.

### Nasal Immunization With Bi-Component Vaccine Formulations Incorporating LAH- and 3M2e-Bearing Nanorings Admixed With MG Promoted the Development of a Potent, Multi-Factorial and Multi-Compartmental Immunity Directed Against Both IAV Epitopes and Protected Mice Against H1N1 Challenge

In light of the promising immunogenicity results obtained with [N-LAH + MG] and [N-3M2e + MG] formulations, we evaluated the protective potential of these preparations administered separately or in combination against a homologous H1N1 challenge in mice.

**Figure 5:**
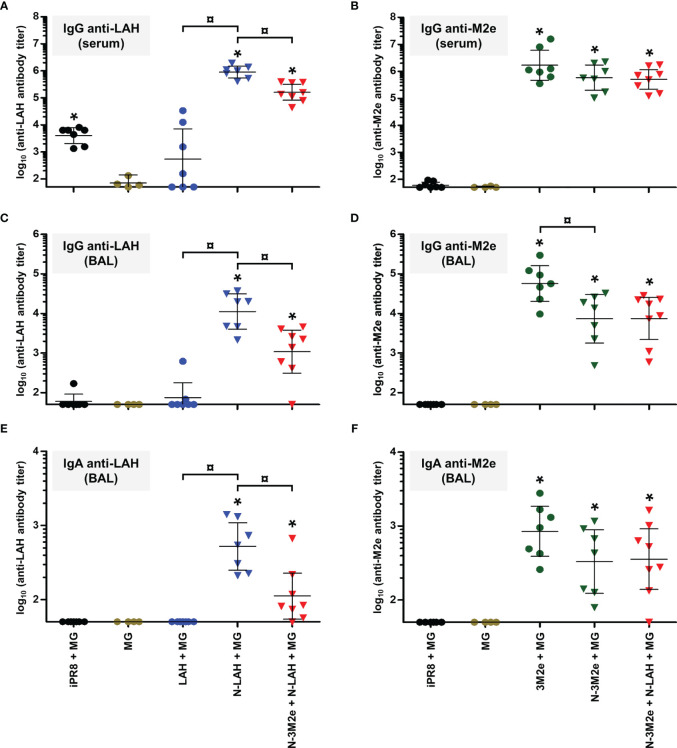
Comparison of specific serum and mucosal humoral responses in mice immunized with LAH- and M2e-bearing nanorings instilled separately or in combination. Mice received at 2-week intervals three intranasal administrations of N-LAH and N-3M2e nanorings admixed with Montanide™ gel (MG) instilled separately or in combination. Control groups received intranasal administrations of free LAH or 3M2e peptides admixed with MG or vaccine vehicle (MG), or subcutaneous administrations of UV-inactivated PR8 strain (iPR8) admixed with MG. Two weeks after the third immunization, **(A)** anti-LAH or **(B)** anti-M2e IgG titers in the serum, and anti-LAH **(C)** IgG and **(E)** IgA or anti-M2e **(D)** IgG and **(F)** IgA titers in the bronchoalveolar lavage fluids (BAL) were determined by ELISA. The titer of each mouse sample is presented, including the geometric mean with 95% confidence interval. *, statistically significant difference (*P <* 0.05) in comparison to MG group. ¤, statistically significant difference (*P <* 0.05) between the indicated groups.

**Figure 6:**
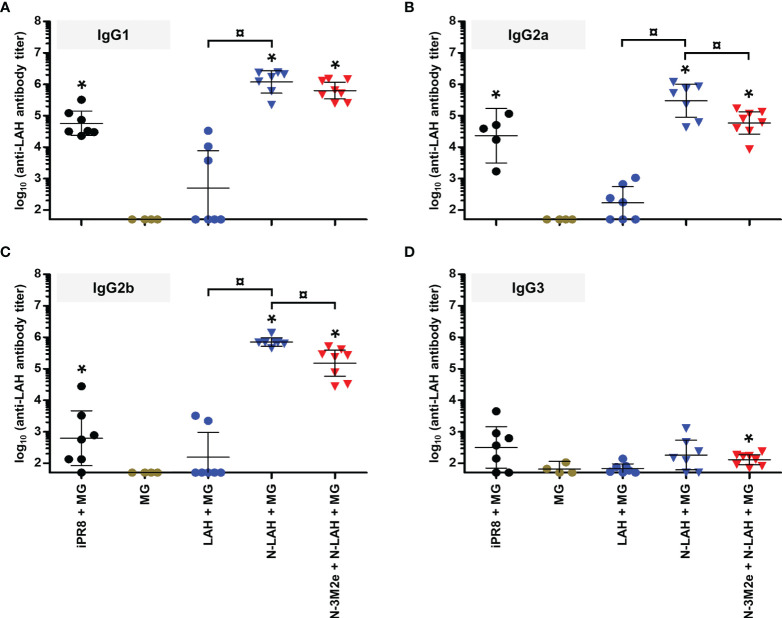
IgG subclass composition of LAH-specific serum humoral response in mice immunized with N-LAH nanorings instilled alone or in combination with N-3M2e nanorings. Mice received at 2-week intervals three intranasal administrations of N-LAH nanorings admixed with Montanide™ gel (MG) instilled alone or in combination with N-3M2e nanorings. Control groups received intranasal administrations of free LAH peptide admixed with MG or vaccine vehicle (MG), or subcutaneous administrations of UV-inactivated PR8 strain (iPR8) admixed with MG. Two weeks after the third immunization, serum anti-LAH **(A)** IgG1, **(B)** IgG2a, **(C)** IgG2b, and **(D)** IgG3 titers were determined by ELISA. The titer of each mouse sample is presented, including the geometric mean with 95% confidence interval. *, statistically significant difference (*P <* 0.05) in comparison to MG group. ¤, statistically significant difference (*P <* 0.05) between the indicated groups.

**Figure 7:**
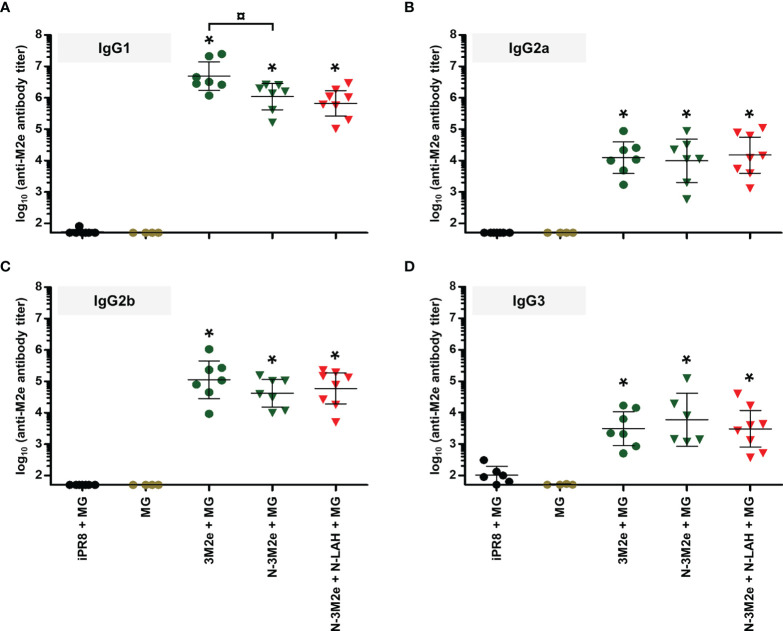
IgG subclass composition of M2e-specific serum humoral response in mice immunized with N-3M2e nanorings instilled alone or in combination with N-LAH nanorings. Mice received at 2-week intervals three intranasal administrations of N-3M2e nanorings admixed with Montanide™ gel (MG) instilled alone or in combination with N-LAH nanorings. Control groups received intranasal administrations of free 3M2e peptide admixed with MG or vaccine vehicle (MG), or subcutaneous administrations of UV-inactivated PR8 strain (iPR8) admixed with MG. Two weeks after the third immunization, serum anti-M2e **(A)** IgG1, **(B)** IgG2a, **(C)** IgG2b, and **(D)** IgG3 titers were determined by ELISA. The titer of each mouse sample is presented, including the geometric mean with 95% confidence interval. *, statistically significant difference (*P <* 0.05) in comparison to MG group. ¤, statistically significant difference (*P <* 0.05) between the indicated groups.

**Figure 8:**
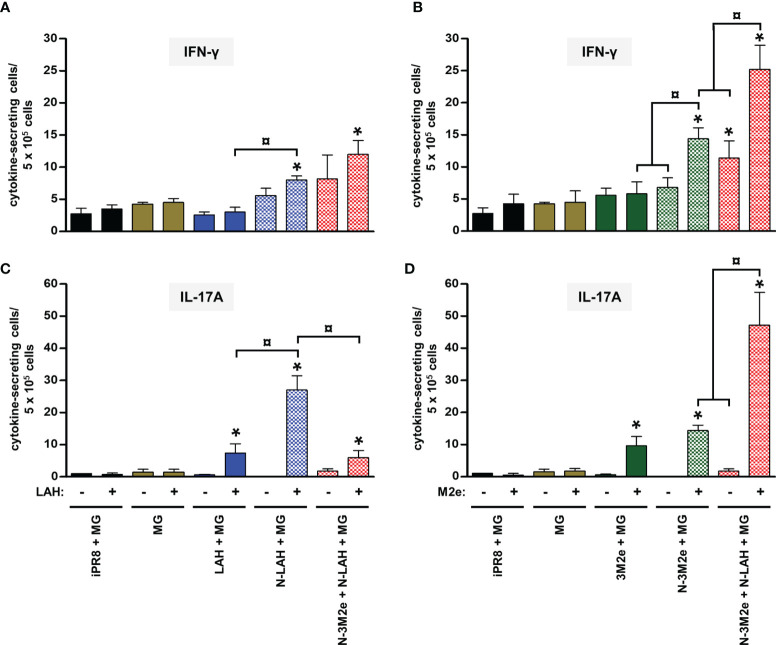
Comparison of specific cellular responses in mice immunized with LAH- and M2e-bearing nanorings instilled separately or in combination. Mice received at 2-week intervals three intranasal administrations of N-LAH and N-3M2e nanorings admixed with Montanide™ gel (MG) instilled separately or in combination. Control groups received intranasal administrations of free LAH or 3M2e peptides admixed with MG or vaccine vehicle (MG), or subcutaneous administrations of UV-inactivated PR8 strain (iPR8) admixed with MG. Two weeks after the third immunization, spleen cells from individual mice were restimulated *ex vivo* for 24 h in presence (‘+’) or absence (‘-’) of synthesized **(A, C)** LAH or **(B, D)** M2e peptide and the frequency of LAH-specific **(A)** IFN-γ- and **(C)** IL-17A-secreting cells or M2e-specific **(B)** IFN-γ- and **(D)** IL-17A-secreting cells was monitored by ELISpot assay. Data are presented as arithmetic means with SEM of 4-5 individual spleens. *, statistically significant difference (*P <* 0.05) in comparison to MG group. ¤, statistically significant difference (*P <* 0.05) between the indicated groups.

Firstly, we compared the characteristics of LAH- and M2e-specific immune responses between mice immunized with [N-3M2e + MG] or [N-LAH + MG] and mice immunized with [N-3M2e + N-LAH + MG]. The addition of N-3M2e nanorings to the [N-LAH + MG] formulation partially reduced the magnitude of LAH-specific systemic **(** **Figure 5A** **)** and local **(** **Figures 5C, E** **)** humoral responses. In contrast, addition of N-3M2e nanorings to the [N-LAH + MG] formulation did not impede the generation of M2e-specific humoral responses **(** **Figures 5B, D, F** **)**. The serum IgG response generated by the chimeric nanorings was composed of both type 1 (IgG2a, IgG2b) **(** **Figures 6B, C**, **7B, C** **)** and type 2 (IgG1) **(** **Figures 6A**, **7A** **)** IgG subclasses. M2e-specific IgG3 titers were also detected in mice immunized with [N-3M2e + MG] and [N-3M2e + N-LAH + MG] **(** **Figure 7D** **)**, and low levels of LAH-specific IgG3 titers were measured in [N-3M2e + N-LAH + MG]-vaccinated mice **(** **Figure 6D** **)**. Mice parenterally vaccinated with [iPR8 + MG] developed a LAH-specific IgG response detectable only in the serum **(** **Figure 5** **)** and composed of both type 1 and type 2 IgG subclasses **(** **Figure 6** **)**, but were unable to mount a M2e-specific humoral response **(** **Figures 5**, **7** **)**. Whereas the addition of N-3M2e nanorings to the [N-LAH + MG] formulation did not impact the generation of LAH-specific IFN-γ-secreting cells **(** **Figure 8A** **)**, it reduced the frequency of LAH-specific IL-17A-secreting cells in the spleen **(** **Figure 8C** **)**. In contrast, the addition of N-LAH nanorings to the [N-3M2e + MG] formulation resulted in an increase in the frequency of M2e-specific IFN-γ- and IL-17A-secreting cells **(** **Figures 8B, D** **)**. The frequency of cells secreting type 2 cytokines was not significantly different between mice immunized with the different preparations and mice instilled with MG after restimulation with LAH or M2e peptides (data not shown).

**Figure 9:**
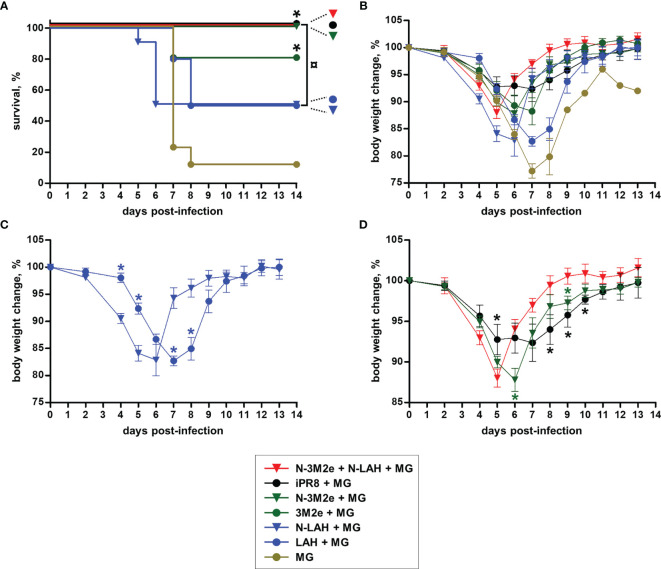
Evaluation of the clinical protection conferred by LAH- and 3M2e-bearing nanorings against homologous H1N1 infection in mice. Mice received at 2-week intervals three intranasal administrations of N-LAH and N-3M2e nanorings admixed with Montanide™ gel (MG) instilled separately or in combination. Experimental control groups received intranasal administrations of free LAH or 3M2e peptides admixed with MG. Mice receiving subcutaneous administrations of UV-inactivated PR8 strain (iPR8) admixed with MG or intranasal administrations of vaccine vehicle (MG) were included as positive or negative control groups, respectively. Two weeks after the third immunization, all mice were challenged with 15 LD_50_ of PR8 strain and monitored daily for **(A)** mortality and **(B–D)** body weight loss. **(A)** Survival curves of infected mice are expressed as the percentages of surviving mice. The log-rank (Mantel-Cox) test was used to compare survival curves. *, statistically significant difference (*P <* 0.05) in comparison to MG group. ¤, statistically significant difference (*P <* 0.05) between the indicated groups. **(B–D)** Weight curves of infected mice are expressed as the arithmetic mean (with SEM) of the percentages of body weight changes between the examined day and the day of infection (day 0). *, statistically significant difference (*P <* 0.05) in comparison to [N-LAH + MG] (panel **C**) or [N-3M2e + N-LAH + MG] groups (panel **D**).

**Figure 10:**
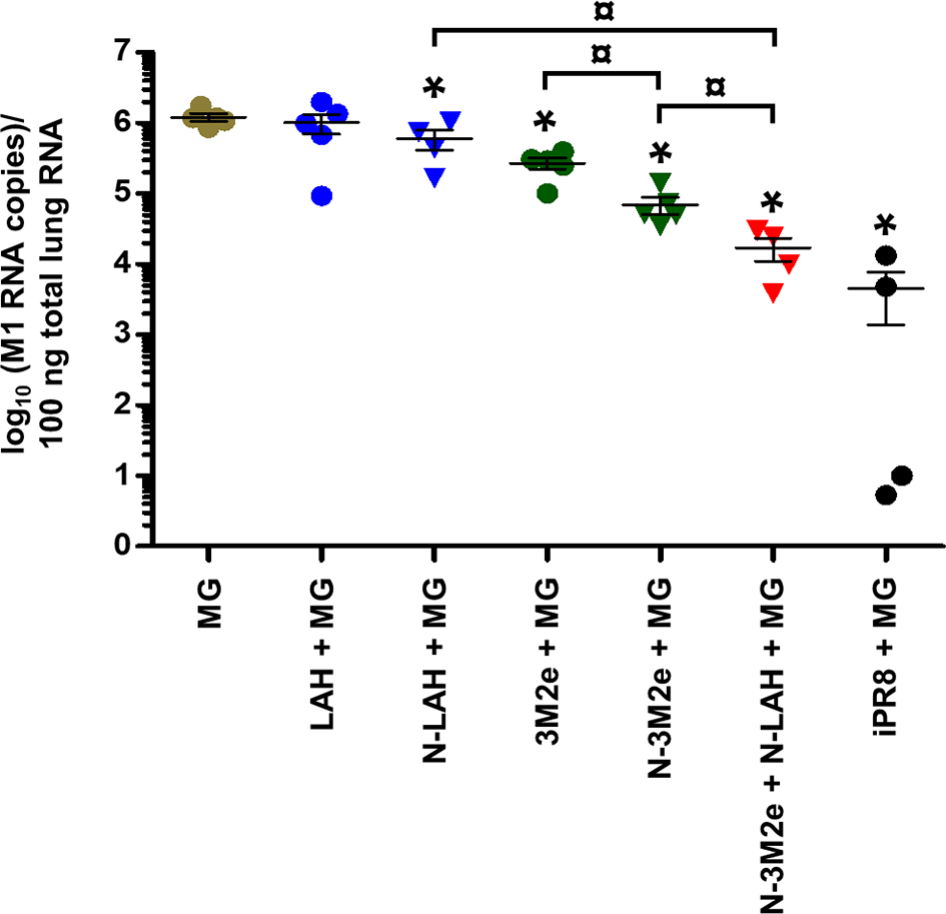
Evaluation of the virological protection conferred by LAH- and 3M2e-bearing nanorings against homologous H1N1 infection in mice. Mice received at 2-week intervals three intranasal administrations of N-LAH and N-3M2e nanorings admixed with Montanide™ gel (MG) instilled separately or in combination. Experimental control groups received intranasal administrations of free LAH or 3M2e peptides admixed with MG. Mice receiving subcutaneous administrations of UV-inactivated PR8 strain (iPR8) admixed with MG or intranasal administrations of vaccine vehicle (MG) were included as positive or negative control groups, respectively. Two weeks after the third immunization, all mice were challenged with 15 LD_50_ of PR8 strain and four days after challenge, mice were sacrificed, and individual viral loads were measured from lung homogenates by qRT-PCR. The number of M1-specific RNA copies for 100 ng total lung RNA was determined against a standard curve using a plasmid encoding PR8 M1 gene. The number of viral copies of each mouse lung sample is presented, including the arithmetic mean with SEM. *, statistically significant difference (*P <* 0.05) in comparison to MG group. ¤, statistically significant difference (*P <* 0.05) between the indicated groups.

Two weeks after the third immunization, mice were challenged with 15 LD_50_ of PR8 strain and monitored daily for mortality **(** **Figure 9A** **)** and weight loss **(** **Figures 9B–D** **)**. Only one out of nine mice instilled with MG survived the infection after having lost 19% of its initial weight at day 6 p.i. In contrast, all mice immunized with [iPR8 + MG] survived the challenge with minor weight loss (mean percentage of initial weight of 92% at day 7 p.i.). The LAH peptide administered free or in fusion with nanorings with MG conferred a partial protection, and a similar clinical picture was observed for both groups (survival rate of 50% in [LAH + MG] and [N-LAH + MG] groups). The 3M2e peptide administered free or in fusion to nanorings conferred a significant protection (survival rates of 80% and 100% in [3M2e + MG] and [N-3M2e + MG] groups, respectively) with little weight loss (mean percentage of initial weight of 88% at day 7 and day 6 p.i. in [3M2e + MG] and [N-3M2e + MG] groups, respectively). Finally, all mice immunized with [N-3M2e + N-LAH + MG] survived infection and presented a transient reduction of weight (mean percentage of initial weight of 88% at day 5 p.i.). These mice showed a faster weight recovery than mice immunized with [N-3M2e + MG] or [iPR8 + MG] between day 6 and day 10 p.i. **(** **Figure 9D** **)**. Virus quantification in the lung homogenates at day 4 p.i. **(** **Figure 10** **)** revealed no major decrease in viral loads between MG-instilled mice and [LAH + MG] or [N-LAH + MG] groups, the latter showing nevertheless statistically lower viral loads than the MG group. In contrast, the administration of [N-3M2e + MG] induced more than 1-log decrease in viral loads in comparison to MG instillation, and statistically less viral copies were measured in the lungs isolated from mice vaccinated with [N-3M2e + MG] than those vaccinated with [3M2e + MG]. Finally, mice immunized with [N-3M2e + N-LAH + MG] displayed an almost 2-log reduction in viral loads *versus* the MG group and significantly less viral copies than mice immunized with [N-3M2e + MG]. Mice which received [iPR8 + MG] had more than 2-log decrease in viral loads *versus* the MG group and there were no statistical differences between [N-3M2e + N-LAH + MG]- and [iPR8 + MG]-immunized groups.

Overall, these results demonstrate that the [N-3M2e + N-LAH + MG] formulation elicited humoral responses composed of serum type 1/type 2 IgG and mucosal IgG and IgA as well as cellular responses dominated by type 1/type 17 cytokine profiles, and conferred clinical and virological protection against H1N1 homologous infection in mice.

### Evaluation of the Immunogenicity and Protective Potential of LAH- and 3M2e-Bearing Chimeric Nanorings Against Heterosubtypic HPAIV H5N8 Infection in Chickens

**Figure 11:**
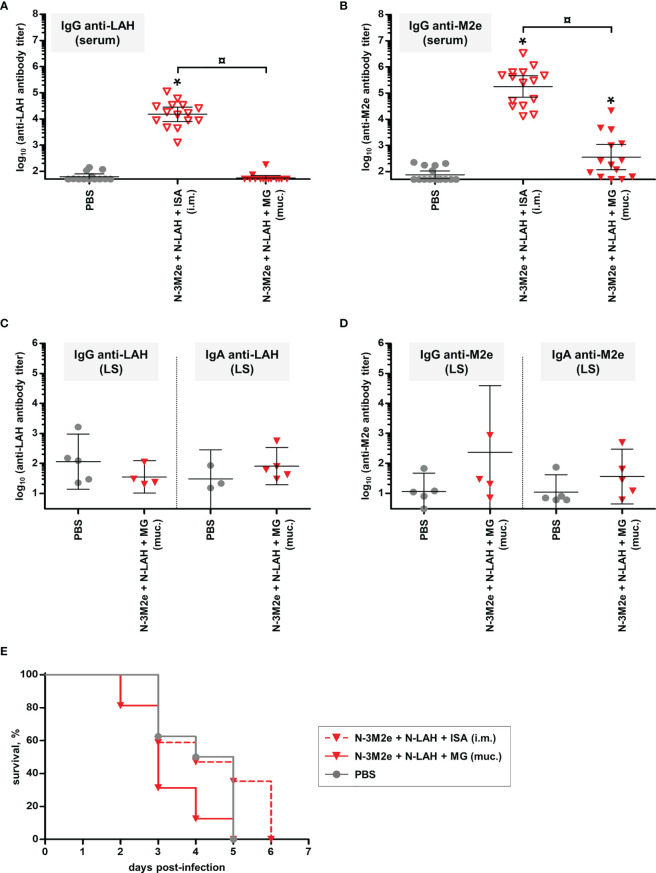
Evaluation of the immunogenicity and protective potential of LAH- and 3M2e-bearing nanorings against heterosubtypic H5N8 HPAIV infection in chickens. Chickens received at 2-week intervals three intranasal administrations of H5N9 LAH and 3M2e peptides fused to nanorings either mucosally (‘muc.’) with Montanide™ gel (MG) or intramuscularly (‘i.m.’) with Montanide™ ISA (ISA). The non-vaccinated control group received only PBS. Eighteen days after the third immunization, serum **(A)** anti-LAH or **(B)** anti-M2e IgG titers or mucosal **(C)** anti-LAH IgG and IgA or **(D)** anti-M2e IgG or IgA titers in the lachrymal secretions (LS) were dosed. The titer of each chicken sample is presented, including the geometric mean with 95% confidence interval. *, statistically significant difference (*P <* 0.05) in comparison to PBS group. ¤, statistically significant difference (*P <* 0.05) between the indicated groups. **(E)** Three weeks after the third immunization, all chickens were challenged with 5 LD_50_ of H5N8 strain and the mortality was monitored daily. Survival curves of infected chickens are expressed as the percentages of surviving chickens.

Experiments in mice have shown that intranasal vaccination of a combination of N-LAH and N-3M2e nanorings admixed with MG generated a protective immunity against homologous IAV infection. In a final set of experiments, we evaluated the immunogenicity of this vaccine formulation in chickens, as well as its cross-protective potential against heterosubtypic HPAIV infection. H5N9 LAH and 3M2e peptides fused to nanorings were administered to birds either mucosally with MG or parenterally with ISA adjuvant. The stability of nanorings mixed with ISA was confirmed previously (38). The non-vaccinated control group received only PBS. Eighteen days after the third immunization, anti-M2e- and anti-LAH antibodies were dosed in the serum **(** **Figures 11A, B** **)** and in the LS **(** **Figures 11C, D** **)** to analyze systemic and mucosal humoral responses, respectively. Chickens immunized with [N-3M2e + N-LAH + ISA] developed LAH- and M2e-specific serum IgG responses. In contrast, [N-3M2e + N-LAH + MG]-immunized birds exhibited a lower, albeit significant, M2e-specific serum IgG response, and no anti-LAH IgG were detected in the serum **(** **Figures 11A, B** **)**. In addition, no significant differences were found in M2e- or LAH-specific IgG or IgA titers in LS between [N-3M2e + N-LAH + MG]- and PBS-instilled chickens **(** **Figures 11C, D** **)**. Three weeks after the third immunization, chickens were challenged with 5 LD_50_ of HPAIV H5N8 strain and the mortality was monitored daily **(** **Figure 11E** **)**. Neither chickens from the [N-3M2e + N-LAH + MG] group, nor chickens from the non-vaccinated group survived the infection (100% mortality within 5 days for both groups). Notwithstanding the presence of elevated specific serum antibody titers, all chickens vaccinated with [N-3M2e + N-LAH + ISA] died within 6 days. No correlation was found between the magnitude of the specific humoral response and the survival time (data not shown). Because of the high mortality rate and the fast kinetics of the infection, pharyngeal or cloacal viral shedding were not analyzed.

## Discussion

Faced with the pressing challenge posed by the high variability associated with IAV which requires surveillance monitoring of circulating strains and regular updating of the composition of current vaccines, novel immunization strategies which include the use of conserved “universal” viral epitopes are urgently needed. In the present study, we evaluated the ability of various intranasal M2e- and LAH-based vaccine formulations to trigger specific antibody- and cell-mediated immunity, two critical adaptive immune arms in the fight against IAV infections, and to confer protection against an experimental IAV challenge in mice and chickens.

M2e and LAH peptides are poorly immunogenic *per se*. Therefore, they have to be administered in association with adjuvant/delivery systems to induce robust immune responses in the host, including ligands of PRRs, bacterial toxins and derivatives, lipid-based particles, virus-like particles, organic and inorganic polymers and bacterial or viral vectored vaccines (5, 7–15). In this study, we have used innovative nanostructures developed in our research unit named “nanorings” as a mucosal vaccine delivery platform. Nanorings are composed of 10-11 N_RSV_ protomers entrapping random stretches of bacterial RNA (36) and are readily internalized by antigen-presenting cells (APCs) (39). Nanorings also stimulate expression of co-stimulatory molecules as well as secretion of type I interferons by APCs (Riffault S., personal communication). We demonstrated that LAH fused to nanorings elicited higher LAH-specific humoral responses, both at systemic and mucosal levels, as well as higher cellular responses than those observed in mice receiving free LAH peptide. The immunopotentiator properties of nanorings may be conferred by the single-stranded bacterial RNA fragment of 70 to 77 bases and/or the nanoparticle architecture of the rings. Such a marked adjuvant effect of nanorings was not observed for the 3M2e epitope, which could be related to our immunization protocol. Indeed, 20 µg of 3M2e peptides contain more M2e epitopes than 20 µg of N-3M2e nanorings (molecular ratio of 1/6 for N-3M2e/3M2e). In addition, mice immunized with N-3M2e nanorings exhibited significantly higher titers of serum anti-M2e IgG than 3M2e-immunized mice after two immunizations (data not shown), as previously described (28), and thus the third immunization may have masked the adjuvant potential of the nanoring platform.

The fusion in tandem of P97c to LAH also boosted immunogenicity of the viral epitope, and the fusion orientation greatly influenced the features of the LAH-specific antibody response. The serum IgG titers were more elevated in the P97c-LAH group than in [LAH-P97c]-instilled mice. Similarly, P97c was shown to enhance the magnitude of the antibody response directed against the capsid protein of the porcine circovirus type 2 only when it was fused at the N-terminal end of the viral epitope (30). A recent *in vitro* study indicated that P97c stimulated innate immune responses through activation of TLR-5 (29). TLR-5 agonists, such as flagellin, are potent and safe adjuvants for influenza vaccines administered *via* parenteral or mucosal routes in both animals and humans. The mucosal adjuvant properties of TLR-5 ligands rely on an enhancement of transepithelial transport of co-administered antigens by the follicle-associated epithelium of the mucosa-associated lymphoid tissues, as well as on the stimulation of the migration of APCs into the follicle-associated epithelium (15). These ligands also stimulate the uptake of vaccine antigens by APCs and promote the activation and the maturation of APCs *in vitro* (15). Nonetheless, P97c remains a poor mucosal adjuvant and low to non-significant specific IgA titers were detected in the BAL of mice immunized with chimeric proteins composed of IAV epitopes fused to P97c either administered alone or in fusion to nanorings. In contrast, N-3M2e and N-LAH nanorings admixed with MG induced a significant mucosal humoral immunity specifically directed against IAV epitopes. MG is a polymeric aqueous adjuvant based on a dispersion of a high-molecular-weight polyacrylic polymer in water whose safety and efficacy have been proven in the context of a mucosal vaccination. However, the mechanisms of action associated with MG are still poorly characterized.

All mice vaccinated with [N-3M2e + N-LAH + MG] survived infection and presented significantly reduced pulmonary viral loads, and various immune defense mechanisms could be engaged. Passive transfer experiments in mice showed that antibodies directed against M2e or LAH mediated resistance against IAV infection (16, 17, 40, 41). An *in vitro* study demonstrated that anti-LAH antibodies exerted broad neutralizing activities against H3 strains and inhibited the conformational change of the HA stalk domain during the fusion step between viral and endosomal membranes (18). However, no functional activity was detected with the serum or BAL of mice vaccinated with our H1 LAH-based formulations using standard microneutralization assay (data not shown). Similarly, a norovirus P particle displaying H1 and H3 LAH epitopes elicited neutralizing antibodies against H3 but not H1 strains, and differences in the conformational flexibility of the two epitopes or in the time of exposure of the epitopes during membrane fusion may explain this result (42, 43). Other effector functions exerted by anti-LAH antibodies may be involved, such as Fc-mediated mechanisms or complement activation (44). Anti-M2e antibodies restricted viral replication by the elimination of infected cells *via* Fc-mediated effector functions mobilizing alveolar macrophages and natural killer cells (7), and both type 1 and type 2 IgG were associated with protection conferred by anti-M2e antibodies (45, 46). Further investigations on the functionality of anti-LAH and anti-M2e antibodies generated by our vaccine preparations are required.

Beside humoral responses, various type 1-, type 2- and type 17-cytokine secreting CD4^+^ (47, 48) and CD8^+^ (49, 50) T cell subsets contribute to anti-IAV immunity by limiting the duration and severity of the disease *via* multiple synergistic effector mechanisms. IAV-specific CD8^+^ T cells are mainly involved in the direct killing of infected cells, and CD4^+^ T cells provide helper functions to B cells and CD8^+^ T cells, regulate the response of innate immune cells, and exert direct cytotoxic functions (51). Both major histocompatibility complex class I- and class II-restricted epitopes are found in LAH and M2e peptides (52–55), and LAH- or M2e-specific CD4^+^ or CD8^+^ T cells secreting type 1, type 2 or type 17 cytokines can be generated by subunit vaccines in mice (40, 54, 56–58). Some studies also indicated a protective role of M2e-specific CD4^+^ T cells (40, 54). We detected LAH- and M2e-specific type 1-, type 2-, and type 17-secreting cells in the spleen of mice immunized with N-LAH and/or N-3M2e nanorings admixed with MG, and the cell subsets engaged in these responses need to be identified.

Various experiments in mice demonstrated that M2e- and LAH-based vaccines conferred better clinical and virological protection against IAV when they were administered mucosally instead of parenterally, and this was correlated with the ability to induce local IgA (28, 59–61). Secretory IgA, which are polymeric IgA produced by B cells in the lamina propria and secreted to mucosal surfaces, are one of the first lines of defense against respiratory pathogens. They neutralize viral infection of respiratory epithelial cells *via* extracellular and intracellular immune exclusion and also exhibit FcαR-mediated effector functions (15, 62). IgA display a broad spectrum of reactivity against heterovariant and heterosubtypic IAV strains, and are thus an essential defensive front line against highly variable IAV (15). Whereas IgA prevent infections of the upper respiratory system, IgG, which are secreted systemically and diffuse in mucosal tissues, are mainly involved in the defense of the lower respiratory tract by decreasing viral pneumonia (15). Mucosal vaccination is also prone to generate tissue-resident memory T cells in the respiratory tract, which was shown to mediate optimal (cross-) resistance against IAV infections (15, 54). Further analyses on the features of the humoral response in the upper respiratory tract, on the functionality of the mucosal antibody response as well as on the characteristics of cellular immunity in the respiratory mucosa of mice immunized with N-LAH and/or N-3M2e admixed with MG are thus necessary.

The protection conferred by the [N-3M2e + N-LAH + MG] preparation in mice was enhanced in comparison to [N-LAH + MG] or [N-3M2e + MG] preparations. Other studies demonstrated that the combination of LAH and M2e epitopes in vaccine preparations increased immunogenic and protective properties (63–65). This is likely due to the cooperation of both LAH- and M2e-specific effector functions. In addition, the supplementation of [N-3M2e + MG] with N-LAH nanorings raised the number of M2e-specific spleen cells secreting type 1 and type 17 cytokines. Memory M2e-specific CD4^+^ T cells have been shown previously to boost the generation of HA-specific IgG responses (54). Additional studies are required to evaluate whether LAH-specific immune mediators can influence the generation of M2e-specific immunity. Ongoing experiments are evaluating the immunogenicity and protective efficacy of chimeric nanorings bearing M2e and LAH epitopes simultaneously (N-3M2e-LAH, N-LAH-3M2e).

In this study, the efficacy of nanoring-based vaccines in chickens was evaluated for the first time. In stark contrast with the potent protective effect conferred by the combination of N-3M2e and N-LAH nanorings in the mouse model, none of the chickens vaccinated with the formulation administered *via* the mucosal or parenteral routes survived a HPAIV infection.

Some studies demonstrated that parenteral or mucosal administrations of M2e epitopes under various vaccine formats, including a fusion protein with TLR agonists, nanoparticles or a bacterial vector, reduced, and sometimes even totally abrogated, viral shedding and the severity of clinical signs or lung lesions after homologous or heterologous LPAIV challenges in chickens (13, 14, 21, 66, 67). The association of M2e with other viral epitopes such as LAH and NA increased the protective potential of these vaccine formulations (12, 68). In contrast, the effectiveness of a stand-alone M2e vaccine remained limited against HPAIV infections (11, 69–71), and only incorporation of other IAV epitopes such as LAH enabled chickens to present significant survival rates (19). Nonetheless, not all chickens immunized with the combination of M2e and LAH epitopes survived the infection and the virus shedding from the respiratory and digestive tracts remained elevated (19). Although vaccinated chickens developed M2e- and/or LAH-specific humoral and cellular immune responses in these studies, the cellular and molecular mechanisms involved, as well as the respective engagement of mucosal and systemic immune compartments in the resistance against LPAIV and HPAIV, remain largely unknown. Further examination of the characteristics and the functionality of the antibody and cell-mediated responses generated in chickens vaccinated with N-LAH and N-3M2e nanorings are needed for a comprehensive interpretation of the lack of protection observed in our study.

While subunit vaccines incorporating universal epitopes did not provide an adequate protection against HPAIV infections, recent studies indicated that M2e and/or LAH-based vaccines improved the (cross-) protective effects of inactivated IAV vaccines in chickens (11, 20, 72). For example, all chickens co-immunized with a recombinant baculovirus expressing LAH, M2e and nucleoprotein epitopes and an H5N1 inactivated vaccine survived a heterologous HPAIV H5N1 challenge and no longer spread the virus in contrast with chickens immunized with the inactivated vaccine alone (72). The supplementation of inactivated IAV vaccines with M2e-based nanoparticles enhanced protection against H5N1 homologous and H5N8 heterologous HPAIV infections (11), as well as against heterologous LPAIV infection (20). Thus, further studies are warranted to evaluate the beneficial effects of N-LAH and N-3M2e nanorings as supplements to current IAV inactivated vaccines in chickens, and especially in broadening the resistance against vaccine-escape viruses.

## Data Availability Statement

The raw data supporting the conclusions of this article will be made available by the authors, without undue reservation.

## Ethics Statement

The animal study was reviewed and approved by Animal Care and Use Committee at the Centre de Recherche de Jouy-en-Josas (COMETHEA) authorization number 2015100910396112v1, APAFIS number 1487 (mouse experiments); European Council Directive CEE86/609 and animal protocols approved by the Ethics Committee “Sciences et santé animale”, committee number 115 (chicken experiments).

## Author Contributions

ChC, DA, SB, RG, and CyC conceived and designed the study. CyC, MM, MT, QV, AM, LS, P-LH, and ChC produced vaccine candidates. CyC, MM, MT, QV, JM, LS, RG, and ChC performed mouse experiments. TF, PB, RV, MD, CyC, MM, and MT performed chicken experiments. CyC and ChC performed data analysis. CyC and ChC wrote the manuscript. CyC, ChC, RG, DA, and SB reviewed and edited the manuscript. All authors contributed and approved the submitted version of the manuscript.

## Funding

This work was supported by a grant from the Livestock Vaccine Innovation Fund (International Development Research Centre, Bill & Melinda Gates Foundation, Global Affairs Canada) to ChC and DA (Project ID 108517).

## Conflict of Interest

The authors declare that the research was conducted in the absence of any commercial or financial relationships that could be construed as a potential conflict of interest.

## Publisher’s Note

All claims expressed in this article are solely those of the authors and do not necessarily represent those of their affiliated organizations, or those of the publisher, the editors and the reviewers. Any product that may be evaluated in this article, or claim that may be made by its manufacturer, is not guaranteed or endorsed by the publisher.

## Supplementary Material

Supplementary Figure 1 Molecular size distribution by volume as detected by dynamic light scattering for the different nanoring preparations. d.nm: hydrodynamic diameter in nanometers.

Supplementary Figure 2 Evaluation of the immunopotentiator effect of nanorings on the frequency of LAH- and M2e-specific IFN-γ-secreting cells in mice. Mice received at 2-week intervals three intranasal administrations of LAH or 3M2e peptides fused to nanorings admixed with Montanide™ gel (MG). Experimental control groups received intranasal administrations of free LAH or 3M2e peptides admixed with MG or subcutaneous administrations of UV-inactivated PR8 strain (iPR8) admixed with MG. Vaccine vehicle (MG) was intranasally administered to negative control group. Two weeks after the third immunization, spleen cells from individual mice were restimulated *ex vivo* for 24 h in presence (‘+’) or absence (‘-’) of synthesized LAH or M2e peptides. The frequency of **(A)** LAH- or **(B)** M2e-specific IFN-γ-secreting cells was monitored by ELISpot assay. Data are presented as arithmetic means with SEM of 4-5 individual spleens. *, statistically significant difference (*P <* 0.05) in comparison to MG group. ¤, statistically significant difference (*P <* 0.05) between the indicated groups.

## References

[1] World Health Organization. Influenza (Avian and Other Zoonotic) (2018). Available at: https://wwwwhoint/news-room/fact-sheets/detail/influenza-(avian-and-other-zoonotic).

[2] Pantin-Jackwood MJ Swayne DE. Pathogenesis and Pathobiology of Avian Influenza Virus Infection in Birds. Rev Sci Tech (2009) 28:113–36. doi: 10.20506/rst.28.1.1869 19618622

[3] Krammer F. The Human Antibody Response to Influenza A Virus Infection and Vaccination. Nat Rev Immunol (2019) 19:383–97. doi: 10.1038/s41577-019-0143-6 30837674

[4] Rajao DS Perez DR. Universal Vaccines and Vaccine Platforms to Protect Against Influenza Viruses in Humans and Agriculture. Front Microbiol (2018) 9:123. doi: 10.3389/fmicb.2018.00123 29467737 PMC5808216

[5] Swayne DE Kapczynski DR. Vaccines and Vaccination for Avian Influenza in Poultry. In: Swayne DE, editor. Animal Influenza. Ames, IA: Wiley-Backwell. (2016). p. 378–434.

[6] Krammer F Palese P. Advances in the Development of Influenza Virus Vaccines. Nat Rev Drug Discovery (2015) 14:167–82. doi: 10.1038/nrd4529 25722244

[7] Kolpe A Schepens B Fiers W Saelens X. M2-Based Influenza Vaccines: Recent Advances and Clinical Potential. Expert Rev Vaccines (2017) 16:123–36. doi: 10.1080/14760584.2017.1240041 27653543

[8] Kostolansky F Tomcikova K Briestenska K Mikusova M Vareckova E. Universal Anti-Influenza Vaccines Based on Viral HA2 and M2e Antigens. Acta Virol (2020) 64:417–26. doi: 10.4149/av_2020_408 33151738

[9] Hajam IA Senevirathne A Hewawaduge C Kim J Lee JH. Intranasally Administered Protein Coated Chitosan Nanoparticles Encapsulating Influenza H9N2 HA2 and M2e mRNA Molecules Elicit Protective Immunity Against Avian Influenza Viruses in Chickens. Vet Res (2020) 51:37. doi: 10.1186/s13567-020-00762-4 32143695 PMC7060564

[10] Watkins HC Pagan CL Childs HR Posada S Chau A Rios J. A Single Dose and Long Lasting Vaccine Against Pandemic Influenza Through the Controlled Release of a Heterospecies Tandem M2 Sequence Embedded Within Detoxified Bacterial Outer Membrane Vesicles. Vaccine (2017) 35:5373–80. doi: 10.1016/j.vaccine.2017.08.013 28866291

[11] Song BM Kang HM Lee EK Jung SC Kim MC Lee YN. Supplemented Vaccination With Tandem Repeat M2e Virus-Like Particles Enhances Protection Against Homologous and Heterologous HPAI H5 Viruses in Chickens. Vaccine (2016) 34:678–86. doi: 10.1016/j.vaccine.2015.11.074 PMC4721577 26691568

[12] Elaish M Xia M Ngunjiri JM Ghorbani A Jang H Kc M. Protective Immunity Against Influenza Virus Challenge by Norovirus P Particle-M2e and HA2-AtCYN Vaccines in Chickens. Vaccine (2019) 37:6454–62. doi: 10.1016/j.vaccine.2019.08.082 31506195

[13] Hajam IA Kim J Lee JH. *Salmonella Gallinarum* Delivering M2eCD40L in Protein and DNA Formats Acts as a Bivalent Vaccine Against Fowl Typhoid and H9N2 Infection in Chickens. Vet Res (2018) 49:99. doi: 10.1186/s13567-018-0593-z 30285855 PMC6389227

[14] Yang WT Yang GL Zhao L Jin YB Jiang YL Huang HB. *Lactobacillus Plantarum* Displaying Conserved M2e and HA2 Fusion Antigens Induces Protection Against Influenza Virus Challenge. Appl Microbiol Biotechnol (2018) 102:5077–88. doi: 10.1007/s00253-018-8924-6 29675804

[15] Calzas C Chevalier C. Innovative Mucosal Vaccine Formulations Against Influenza A Virus Infections. Front Immunol (2019) 10:1605. doi: 10.3389/fimmu.2019.01605 31379823 PMC6650573

[16] Wang TT Tan GS Hai R Pica N Ngai L Ekiert DC. Vaccination With a Synthetic Peptide From the Influenza Virus Hemagglutinin Provides Protection Against Distinct Viral Subtypes. Proc Natl Acad Sci USA (2010) 107:18979–84. doi: 10.1073/pnas.1013387107 PMC2973924 20956293

[17] Chen S Zheng D Li C Zhang W Xu W Liu X. Protection Against Multiple Subtypes of Influenza Viruses by Virus-Like Particle Vaccines Based on a Hemagglutinin Conserved Epitope. BioMed Res Int (2015) 2015:901817. doi: 10.1155/2015/901817 25767809 PMC4341857

[18] Gong X Yin H Shi Y Guan S He X Yang L. Conserved Stem Fragment From H3 Influenza Hemagglutinin Elicits Cross-Clade Neutralizing Antibodies Through Stalk-Targeted Blocking of Conformational Change During Membrane Fusion. Immunol Lett (2016) 172:11–20. doi: 10.1016/j.imlet.2016.02.006 26875772

[19] Li J Helal Z Ladman B Karch C Gelb J Burkhard P. Nanoparticle Vaccine for Avian Influenza Virus: A Challenge Study Against Highly Pathogenic H5N2 Subtype. J Virol Antivir Res (2018) 7:1. doi: 10.4172/2324-8955.1000179

[20] Elaish M Ngunjiri JM Ali A Xia M Ibrahim M Jang H. Supplementation of Inactivated Influenza Vaccine With Norovirus P Particle-M2e Chimeric Vaccine Enhances Protection Against Heterologous Virus Challenge in Chickens. PloS One (2017) 12:e0171174. doi: 10.1371/journal.pone.0171174 28151964 PMC5289506

[21] Elaish M Kang KI Xia M Ali A Shany SA Wang L. Immunogenicity and Protective Efficacy of the Norovirus P Particle-M2e Chimeric Vaccine in Chickens. Vaccine (2015) 33:4901–9. doi: 10.1016/j.vaccine.2015.07.049 26232342

[22] Hoft DF Lottenbach KR Blazevic A Turan A Blevins TP Pacatte TP. Comparisons of the Humoral and Cellular Immune Responses Induced by Live Attenuated Influenza Vaccine and Inactivated Influenza Vaccine in Adults. Clin Vaccine Immunol (2017) 24:e00414–16. doi: 10.1128/CVI.00414-16 PMC5216430 27847366

[23] Zens KD Chen JK Farber DL. Vaccine-Generated Lung Tissue-Resident Memory T Cells Provide Heterosubtypic Protection to Influenza Infection. JCI Insight (2016) 1:e85832. doi: 10.1172/jci.insight.85832 PMC4959801 27468427

[24] Wang T Wei F Liu J. Emerging Role of Mucosal Vaccine in Preventing Infection With Avian Influenza A Viruses. Viruses (2020) 12:862. doi: 10.3390/v12080862 PMC7472103 32784697

[25] Blanco-Lobo P Nogales A Rodriguez L Martinez-Sobrido L. Novel Approaches for the Development of Live Attenuated Influenza Vaccines. Viruses (2019) 11:190. doi: 10.3390/v11020190 PMC6409754 30813325

[26] Sarawar S Hatta Y Watanabe S Dias P Neumann G Kawaoka Y. M2SR, a Novel Live Single Replication Influenza Virus Vaccine, Provides Effective Heterosubtypic Protection in Mice. Vaccine (2016) 34:5090–8. doi: 10.1016/j.vaccine.2016.08.061 PMC5038585 27595896

[27] Jang YH Seong BL. Immune Responses Elicited by Live Attenuated Influenza Vaccines as Correlates of Universal Protection Against Influenza Viruses. Vaccines (Basel) (2021) 9:353. doi: 10.3390/vaccines9040353 33916924 PMC8067561

[28] Herve PL Raliou M Bourdieu C Dubuquoy C Petit-Camurdan A Bertho N. A Novel Subnucleocapsid Nanoplatform for Mucosal Vaccination Against Influenza Virus That Targets the Ectodomain of Matrix Protein 2. J Virol (2014) 88:325–38. doi: 10.1128/JVI.01141-13 PMC3911713 24155388

[29] Gauthier L Babych M Segura M Bourgault S Archambault D. Identification of a Novel TLR5 Agonist Derived From the P97 Protein of *Mycoplasma Hyopneumoniae* . Immunobiol (2020) 225:151962. doi: 10.1016/j.imbio.2020.151962 32747018

[30] Roques E Girard A Gagnon CA Archambault D. Antibody Responses Induced in Mice Immunized With Recombinant Adenovectors Expressing Chimeric Proteins of Various Porcine Pathogens. Vaccine (2013) 31:2698–704. doi: 10.1016/j.vaccine.2013.03.068 23583895

[31] Tran TL Castagne N Bhella D Varela PF Bernard J Chilmonczyk S. The Nine C-Terminal Amino Acids of the Respiratory Syncytial Virus Protein P Are Necessary and Sufficient for Binding to Ribonucleoprotein Complexes in Which Six Ribonucleotides Are Contacted Per N Protein Protomer. J Gen Virol (2007) 88:196–206. doi: 10.1099/vir.0.82282-0 17170452

[32] Castagne N Barbier A Bernard J Rezaei H Huet JC Henry C. Biochemical Characterization of the Respiratory Syncytial Virus P-P and P-N Protein Complexes and Localization of the P Protein Oligomerization Domain. J Gen Virol (2004) 85:1643–53. doi: 10.1099/vir.0.79830-0 15166449

[33] Ganapathy K Cargill PW. Jones RC. A Comparison of Methods of Inducing Lachrymation and Tear Collection in Chickens for Detection of Virus-Specific Immuoglobulins After Infection With Infectious Bronchitis Virus. Avian Pathol (2005) 34:248–51. doi: 10.1080/03079450500112344 16191709

[34] Calzas C Goyette-Desjardins G Lemire P Gagnon F Lachance C Van Calsteren MR. Group B *Streptococcus* and *Streptococcus Suis* Capsular Polysaccharides Induce Chemokine Production by Dendritic Cells *via* Toll-Like Receptor 2- and MyD88-Dependent and -Independent Pathways. Infect Immun (2013) 81:3106–18. doi: 10.1128/IAI.00113-13 PMC3754219 23774593

[35] Le Goffic R Bouguyon E Chevalier C Vidic J Da Costa B Leymarie O. Influenza A Virus Protein PB1-F2 Exacerbates IFN-Beta Expression of Human Respiratory Epithelial Cells. J Immunol (2010) 185:4812–23. doi: 10.4049/jimmunol.0903952 20844191

[36] Tawar RG Duquerroy S Vonrhein C Varela PF Damier-Piolle L Castagne N. Crystal Structure of a Nucleocapsid-Like Nucleoprotein-RNA Complex of Respiratory Syncytial Virus. Science (2009) 326:1279–83. doi: 10.1126/science.1177634 19965480

[37] Murakami M Kamimura D Hirano T. Pleiotropy and Specificity: Insights From the Interleukin 6 Family of Cytokines. Immunity (2019) 50:812–31. doi: 10.1016/j.immuni.2019.03.027 30995501

[38] Riffault S Meyer G Deplanche M Dubuquoy C Durand G Soulestin M. A New Subunit Vaccine Based on Nucleoprotein Nanoparticles Confers Partial Clinical and Virological Protection in Calves Against Bovine Respiratory Syncytial Virus. Vaccine (2010) 28:3722–34. doi: 10.1016/j.vaccine.2010.03.008 PMC7115569 20307593

[39] Roux X Dubuquoy C Durand G Tran-Tolla TL Castagne N Bernard J. Sub-Nucleocapsid Nanoparticles: A Nasal Vaccine Against Respiratory Syncytial Virus. PloS One (2008) 3:e1766. doi: 10.1371/journal.pone.0001766 18335041 PMC2262139

[40] Lee YN Lee YT Kim MC Hwang HS Lee JS Kim KH. Fc Receptor Is Not Required for Inducing Antibodies But Plays a Critical Role in Conferring Protection After Influenza M2 Vaccination. Immunology (2014) 143:300–9. doi: 10.1111/imm.12310 PMC4172145 24773389

[41] Neirynck S Deroo T Saelens X Vanlandschoot P Jou WM Fiers W. A Universal Influenza A Vaccine Based on the Extracellular Domain of the M2 Protein. Nat Med (1999) 5:1157–63. doi: 10.1038/13484 10502819

[42] Gong X Yin H Shi Y He X Yu Y Guan S. Evaluation of the Immunogenicity and Protective Effects of a Trivalent Chimeric Norovirus P Particle Immunogen Displaying Influenza HA2 From Subtypes H1, H3 and B. Emerg Microbes Infect (2016) 5:e51. doi: 10.1038/emi.2016.51 27222326 PMC4893548

[43] Mallajosyula VV Citron M Lu X Meulen JT Varadarajan R Liang X. *In Vitro* and *In Vivo* Characterization of Designed Immunogens Derived From the CD-Helix of the Stem of Influenza Hemagglutinin. Proteins (2013) 81:1759–75. doi: 10.1002/prot.24317 23625724

[44] Tomcikova K Vareckova E. Different Mechanisms of the Protection Against Influenza A Infection Mediated by Broadly Reactive HA2-Specific Antibodies. Acta Virol (2019) 63:347–65. doi: 10.4149/av_2019_408 31802678

[45] Mozdzanowska K Zharikova D Cudic M Otvos L Gerhard W. Roles of Adjuvant and Route of Vaccination in Antibody Response and Protection Engendered by a Synthetic Matrix Protein 2-Based Influenza A Virus Vaccine in the Mouse. Virol J (2007) 4:118. doi: 10.1186/1743-422X-4-118 17974006 PMC2186315

[46] El Bakkouri K Descamps F De Filette M Smet A Festjens E Birkett A. Universal Vaccine Based on Ectodomain of Matrix Protein 2 of Influenza A: Fc Receptors and Alveolar Macrophages Mediate Protection. J Immunol (2011) 186:1022–31. doi: 10.4049/jimmunol.0902147 21169548

[47] Brown DM Lee S Garcia-Hernandez Mde L Swain SL. Multifunctional CD4 Cells Expressing Gamma Interferon and Perforin Mediate Protection Against Lethal Influenza Virus Infection. J Virol (2012) 86:6792–803. doi: 10.1128/JVI.07172-11 PMC3393557 22491469

[48] McKinstry KK Strutt TM Buck A Curtis JD Dibble JP Huston G. IL-10 Deficiency Unleashes an Influenza-Specific Th17 Response and Enhances Survival Against High-Dose Challenge. J Immunol (2009) 182:7353–63. doi: 10.4049/jimmunol.0900657 PMC2724021 19494257

[49] Cerwenka A Morgan TM Harmsen AG Dutton RW. Migration Kinetics and Final Destination of Type 1 and Type 2 CD8 Effector Cells Predict Protection Against Pulmonary Virus Infection. J Exp Med (1999) 189:423–34. doi: 10.1084/jem.189.2.423 PMC2192982 9892624

[50] Hamada H Garcia-Hernandez Mde L Reome JB Misra SK Strutt TM McKinstry KK. Tc17, a Unique Subset of CD8 T Cells That Can Protect Against Lethal Influenza Challenge. J Immunol (2009) 182:3469–81. doi: 10.4049/jimmunol.0801814 PMC2667713 19265125

[51] Jansen JM Gerlach T Elbahesh H Rimmelzwaan GF Saletti G. Influenza Virus-Specific CD4+ and CD8+ T Cell-Mediated Immunity Induced by Infection and Vaccination. J Clin Virol (2019) 119:44–52. doi: 10.1016/j.jcv.2019.08.009 31491709

[52] Gianfrani C Oseroff C Sidney J Chesnut RW Sette A. Human Memory CTL Response Specific for Influenza A Virus Is Broad and Multispecific. Hum Immunol (2000) 61:438–52. doi: 10.1016/s0198-8859(00)00105-1 10773346

[53] Jameson J Cruz J Ennis FA. Human Cytotoxic T-Lymphocyte Repertoire to Influenza A Viruses. J Virol (1998) 72:8682–9. doi: 10.1128/JVI.72.11.8682-8689.1998 PMC110281 9765409

[54] Eliasson DG Omokanye A Schon K Wenzel UA Bernasconi V Bemark M. M2e-Tetramer-Specific Memory CD4 T Cells Are Broadly Protective Against Influenza Infection. Mucosal Immunol (2018) 11:273–89. doi: 10.1038/mi.2017.14 28295019

[55] Stanekova Z Adkins I Kosova M Janulikova J Sebo P Vareckova E. Heterosubtypic Protection Against Influenza A Induced by Adenylate Cyclase Toxoids Delivering Conserved HA2 Subunit of Hemagglutinin. Antiviral Res (2013) 97:24–35. doi: 10.1016/j.antiviral.2012.09.008 23036818

[56] Stepanova LA Kotlyarov RY Kovaleva AA Potapchuk MV Korotkov AV Sergeeva MV. Protection Against Multiple Influenza A Virus Strains Induced by Candidate Recombinant Vaccine Based on Heterologous M2e Peptides Linked to Flagellin. PloS One (2015) 10:e0119520. doi: 10.1371/journal.pone.0119520 25799221 PMC4370815

[57] Lee YN Kim MC Lee YT Hwang HS Cho MK Lee JS. AS04-Adjuvanted Virus-Like Particles Containing Multiple M2 Extracellular Domains of Influenza Virus Confer Improved Protection. Vaccine (2014) 32:4578–85. doi: 10.1016/j.vaccine.2014.06.040 PMC4126506 24951867

[58] Stepanova LA Mardanova ES Shuklina MA Blokhina EA Kotlyarov RY Potapchuk MV. Flagellin-Fused Protein Targeting M2e and HA2 Induces Potent Humoral and T-Cell Responses and Protects Mice Against Various Influenza Viruses A Subtypes. J BioMed Sci (2018) 25:33. doi: 10.1186/s12929-018-0433-5 29631629 PMC5891888

[59] De Filette M Ramne A Birkett A Lycke N Lowenadler B Min Jou W. The Universal Influenza Vaccine M2e-HBc Administered Intranasally in Combination With the Adjuvant CTA1-DD Provides Complete Protection. Vaccine (2006) 24:544–51. doi: 10.1016/j.vaccine.2005.08.061 16169634

[60] Zheng D Chen S Qu D Chen J Wang F Zhang R. Influenza H7N9 LAH-HBc Virus-Like Particle Vaccine With Adjuvant Protects Mice Against Homologous and Heterologous Influenza Viruses. Vaccine (2016) 34:6464–71. doi: 10.1016/j.vaccine.2016.11.026 27866773

[61] Qi M Zhang XE Sun X Zhang X Yao Y Liu S. Intranasal Nanovaccine Confers Homo- and Hetero-Subtypic Influenza Protection. Small (2018) 14:e1703207. doi: 10.1002/smll.201703207 29430819

[62] Mullarkey CE Bailey MJ Golubeva DA Tan GS Nachbagauer R He W. Broadly Neutralizing Hemagglutinin Stalk-Specific Antibodies Induce Potent Phagocytosis of Immune Complexes by Neutrophils in an Fc-Dependent Manner. mBio (2016) 7:e01624–16. doi: 10.1128/mBio.01624-16 PMC5050345 27703076

[63] Ni Y Guo J Turner D Tizard I. Development of a Novel Dual-Domain Nanoparticle Antigen Construct for Universal Influenza Vaccine. Vaccine (2017) 35:7026–32. doi: 10.1016/j.vaccine.2017.10.051 PMC5715465 29102171

[64] Tsybalova LM Stepanova LA Shuklina MA Mardanova ES Kotlyarov RY Potapchuk MV. Combination of M2e Peptide With Stalk HA Epitopes of Influenza A Virus Enhances Protective Properties of Recombinant Vaccine. PloS One (2018) 13:e0201429. doi: 10.1371/journal.pone.0201429 30138320 PMC6107133

[65] Deng L Kim JR Chang TZ Zhang H Mohan T Champion JA. Protein Nanoparticle Vaccine Based on Flagellin Carrier Fused to Influenza Conserved Epitopes Confers Full Protection Against Influenza A Virus Challenge. Virology (2017) 509:82–9. doi: 10.1016/j.virol.2017.06.001 PMC5526067 28622575

[66] Babapoor S Neef T Mittelholzer C Girshick T Garmendia A Shang H. A Novel Vaccine Using Nanoparticle Platform to Present Immunogenic M2e Against Avian Influenza Infection. Influenza Res Treat (2011) 2011:126794. doi: 10.1155/2011/126794 23074652 PMC3447297

[67] Dabaghian M Latify AM Tebianian M Nili H Ranjbar AR Mirjalili A. Vaccination With Recombinant 4 X M2e.HSP70c Fusion Protein as a Universal Vaccine Candidate Enhances Both Humoral and Cell-Mediated Immune Responses and Decreases Viral Shedding Against Experimental Challenge of H9N2 Influenza in Chickens. Vet Microbiol (2014) 174:116–26. doi: 10.1016/j.vetmic.2014.09.009 25293397

[68] Kim JH Hajam IA Lee JH. Oral Immunization With a Novel Attenuated *Salmonella Typhimurium* Encoding Influenza HA, M2e and NA Antigens Protects Chickens Against H7N9 Infection. Vet Res (2018) 49:12. doi: 10.1186/s13567-018-0509-y 29391053 PMC5796500

[69] Zhang X Liu M Liu C Du J Shi W Sun E. Vaccination With Different M2e Epitope Densities Confers Partial Protection Against H5N1 Influenza A Virus Challenge in Chickens. Intervirology (2011) 54:290–9. doi: 10.1159/000319440 21228535

[70] Reese KA Lupfer C Johnson RC Mitev GM Mullen VM Geller BL. A Novel Lactococcal Vaccine Expressing a Peptide From the M2 Antigen of H5N2 Highly Pathogenic Avian Influenza A Virus Prolongs Survival of Vaccinated Chickens. Vet Med Int (2013) 2013:316926. doi: 10.1155/2013/316926 23766929 PMC3674685

[71] Layton SL Kapczynski DR Higgins S Higgins J Wolfenden AD Liljebjelke KA. Vaccination of Chickens With Recombinant Salmonella Expressing M2e and CD154 Epitopes Increases Protection and Decreases Viral Shedding After Low Pathogenic Avian Influenza Challenge. Poult Sci (2009) 88:2244–52. doi: 10.3382/ps.2009-00251 19834072

[72] Zhang Z Zhang J Zhang J Li Q Miao P Liu J. Coimmunization With Recombinant Epitope-Expressing Baculovirus Enhances Protective Effects of Inactivated H5N1 Vaccine Against Heterologous Virus. Vet Microbiol (2017) 203:143–8. doi: 10.1016/j.vetmic.2017.03.004 28619136

