# Supplementary material for: Immunogenicity and Protective Potential of Mucosal Vaccine Formulations Based on Conserved Epitopes of Influenza A Viruses Fused to an Innovative Ring Nanoplatform in Mice and Chickens
Source: Front Immunol. 2021 Nov 11;12:772550. doi: 10.3389/fimmu.2021.772550 (PMC8632632; doi:10.3389/fimmu.2021.772550)
Supplement: Supplementary Figure 1 — Molecular size distribution by volume as detected by dynamic light scattering for the different nanoring preparations. d.nm: hydrodynamic diameter in nanometers. [file Presentation_1.pptx]

## Slide 1
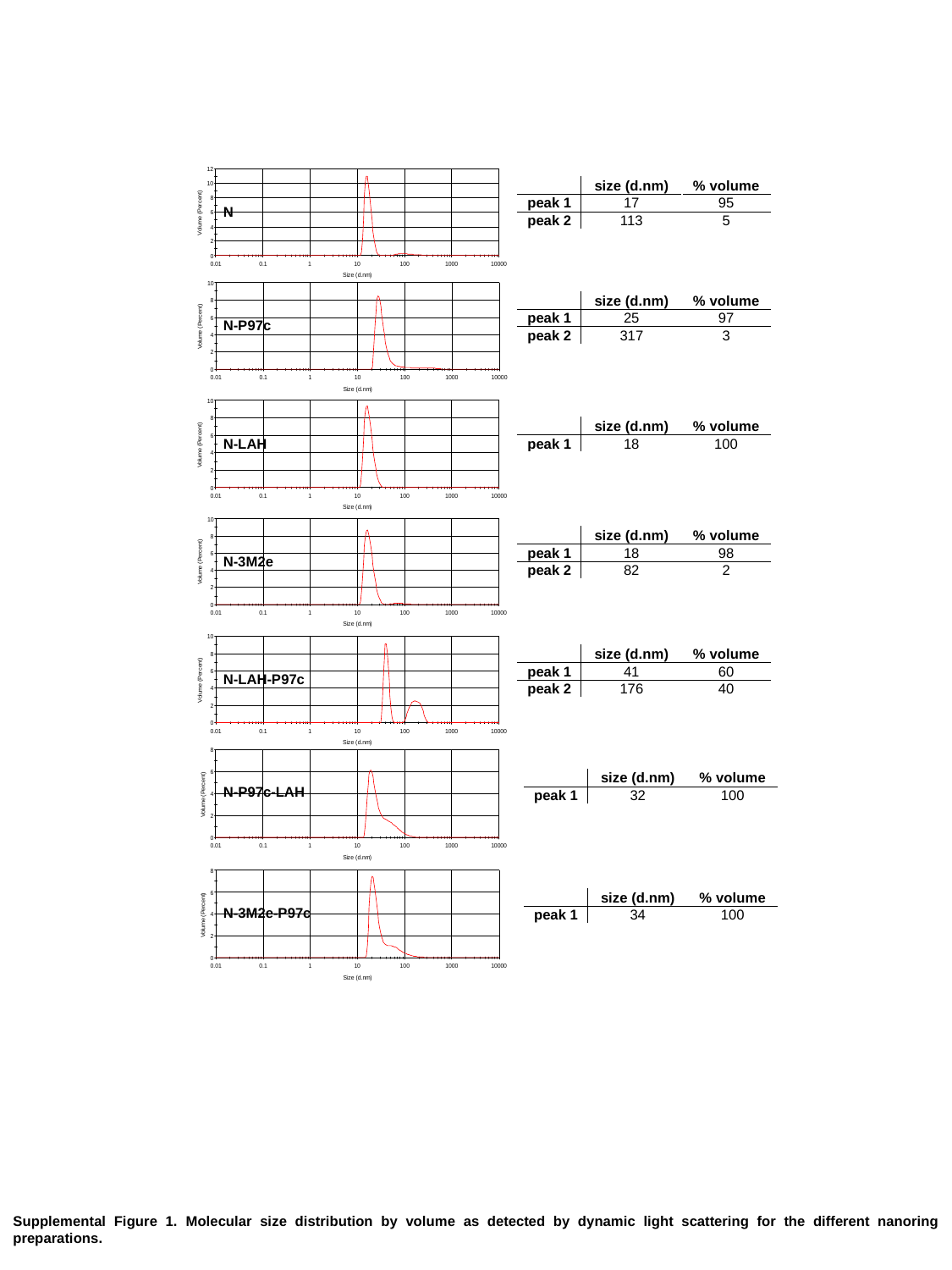

| | size (d.nm) | % volume |
| --- | --- | --- |
| peak 1 | 17 | 95 |
| peak 2 | 113 | 5 |
N
| | size (d.nm) | % volume |
| --- | --- | --- |
| peak 1 | 25 | 97 |
| peak 2 | 317 | 3 |
N-P97c
| | size (d.nm) | % volume |
| --- | --- | --- |
| peak 1 | 18 | 100 |
N-LAH
| | size (d.nm) | % volume |
| --- | --- | --- |
| peak 1 | 18 | 98 |
| peak 2 | 82 | 2 |
N-3M2e
| | size (d.nm) | % volume |
| --- | --- | --- |
| peak 1 | 41 | 60 |
| peak 2 | 176 | 40 |
N-LAH-P97c
| | size (d.nm) | % volume |
| --- | --- | --- |
| peak 1 | 32 | 100 |
N-P97c-LAH
| | size (d.nm) | % volume |
| --- | --- | --- |
| peak 1 | 34 | 100 |
N-3M2e-P97c
Supplemental Figure 1. Molecular size distribution by volume as detected by dynamic light scattering for the different nanoring preparations.

## Slide 2
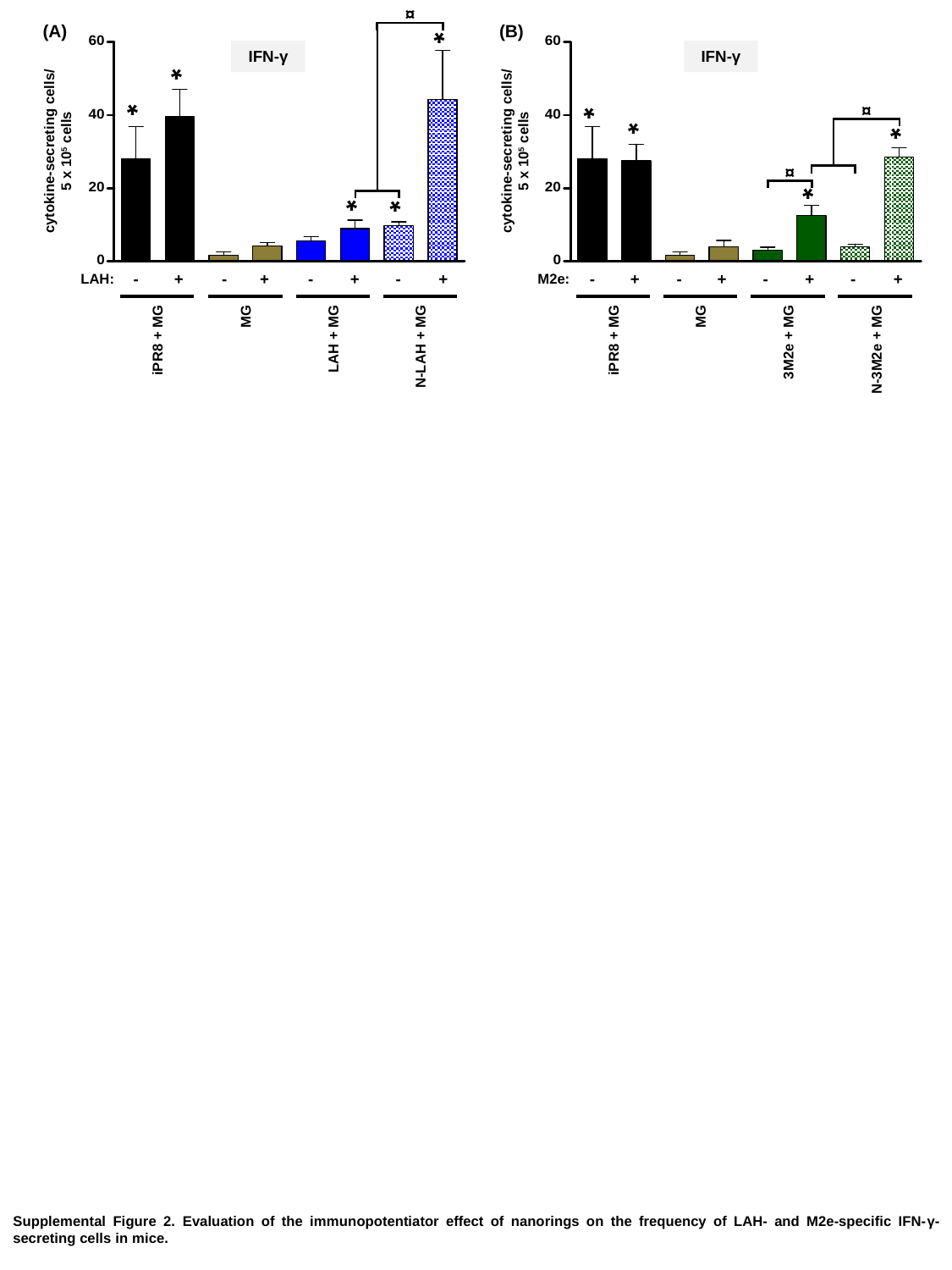

¤
(A)
(B)
*
IFN-γ
IFN-γ
*
*
*
¤
*
*
cytokine-secreting cells/
5 x 10^5^ cells
cytokine-secreting cells/
5 x 10^5^ cells
¤
*
*
*
-
+
-
+
-
+
-
+
LAH:
iPR8 + MG
MG
LAH + MG
N-LAH + MG
-
+
-
+
-
+
-
+
M2e:
iPR8 + MG
MG
3M2e + MG
N-3M2e + MG
Supplemental Figure 2. Evaluation of the immunopotentiator effect of nanorings on the frequency of LAH- and M2e-specific IFN-γ-secreting cells in mice.
